# Efficacy of dehydroepiandrosterone priming in women with poor ovarian response undergoing IVF/ICSI: a meta-analysis

**DOI:** 10.3389/fendo.2023.1156280

**Published:** 2023-06-09

**Authors:** Jie Zhang, Hongyan Jia, Feiyang Diao, Xiang Ma, Jiayin Liu, Yugui Cui

**Affiliations:** ^1^ State Key Laboratory of Reproductive Medicine, Clinical Center of Reproductive Medicine, The First Affiliated Hospital of Nanjing Medical University, Nanjing, China; ^2^ Clinical Centre of Reproductive Medicine, Lianyungang Maternal and Child Health Hospital Kangda College of Nanjing Medical University, Lianyungang, China

**Keywords:** dehydroepiandrosterone, diminished ovarian reserve, poor ovarian response, fertilization *in vitro*, pregnancy outcome

## Abstract

**Background:**

Dehydroepiandrosterone (DHEA) may improve the outcomes of patients with poor ovarian response (POR) or diminished ovarian reserve (DOR) undergoing IVF/ICSI. However, the evidence remains inconsistent. This study aimed to investigate the efficacy of DHEA supplementation in patients with POR/DOR undergoing IVF/ICSI.

**Methods:**

PubMed, Web of Science, Cochrane Library, China National Knowledge Infrastructure (CNKI) were searched up to October 2022.

**Results:**

A total of 32 studies were retrieved, including 14 RCTs, 11 self-controlled studies and 7 case-controlled studies. In the subgroup analysis of only RCTs, DHEA treatment significantly increased the number of antral follicle count (AFC) (weighted mean difference : WMD 1.18, 95% confidence interval(CI): 0.17 to 2.19, *P=*0.022), while reduced the level of bFSH (WMD -1.99, 95% CI: -2.52 to -1.46, *P*<0.001), the need of gonadotropin (Gn) doses (WMD -382.29, 95% CI: -644.82 to -119.76, *P=*0.004), the days of stimulation (WMD -0.90, 95% CI: -1.34 to -0.47, *P* <0.001) and miscarriage rate (relative risk : RR 0.46, 95% CI: 0.29 to 0.73, *P=*0.001). The higher clinical pregnancy and live birth rates were found in the analysis of non-RCTs. However, there were no significant differences in the number of retrieved oocytes, the number of transferred embryos, and the clinical pregnancy and live birth rates in the subgroup analysis of only RCTs. Moreover, meta-regression analyses showed that women with lower basal FSH had more increase in serum FSH levels (b=-0.94, 95% CI: -1.62 to -0.25, *P=*0.014), and women with higher baseline AMH levels had more increase in serum AMH levels (b=-0.60, 95% CI: -1.15 to -0.06, *P=*0.035) after DHEA supplementation. In addition, the number of retrieved oocytes was higher in the studies on relatively younger women (b=-0.21, 95% CI: -0.39 to -0.03, *P=*0.023) and small sample sizes (b=-0.003, 95% CI: -0.006 to -0.0003, *P=*0.032).

**Conclusions:**

DHEA treatment didn’t significantly improve the live birth rate of women with DOR or POR undergoing IVF/ICSI in the subgroup analysis of only RCTs. The higher clinical pregnancy and live birth rates in those non-RCTs should be interpreted with caution because of potential bias. Further studies using more explicit criteria to subjects are needed.

**Systematic review registration:**

https://www.crd.york.ac.uk/prospero/, identifier CRD 42022384393.

## 1 Introduction

Given advancements in assisted reproductive technology (ART), poor ovarian response (POR) or diminished ovarian reserve (DOR) remains the most challenging problem in clinical practice. The incidence of POR varies from 9-24% among patients undergoing ovarian stimulation for *in vitro* fertilization (IVF) or intracytoplasmic sperm injection (ICSI) (1). It’s known that the number of oocytes retrieved is the major determinant of outcomes in ARTs; the higher the number of oocytes retrieved, the higher the cumulative live-birth rate (2). However, women with POR often respond poorly to ovarian stimulation, resulting in fewer oocytes, low implantation rate and pregnancy rates (2-4%), and high cycle cancellation rates (20%) (3, 4). Over the years of ART development, various methods were proposed to improve the cycle outcome of POR women, however, most of them were not showed the satisfactory evidence to be recommended.

Dehydroepiandrosterone (DHEA), an endogenous steroid, is originated from the adrenal glands (80%) and ovarian theca cells (20%) (5). DHEA plays the androgen’s roles by converting into testosterone and dihydrotestosterone in the targeted organs. A plenty of animal studies showed that DHEA supplementation could stimulate granulosa cell proliferation, amplify responsiveness to FSH and in turn stimulate the recruitability of preantral and antral follicles (6, 7). Besides laboratory evidence, clinical observations found that those women with polycystic ovary syndrome (PCOS), congenital adrenal hyperplasia and female-to-male transgender often exhibited higher density of primordial and preantral follicles in their ovaries (8), which further suggested that exposure to more androgens may lead to the increased number of developing follicles and the improved outcomes of IVF/ICSI.

In the last decades, many studies have investigated the efficacy of DHEA priming in women with POR. However, the evidence of clinical outcomes remains insufficient, or even contradictory. There was large heterogeneity in related studies, such as the definitions of PORs, baseline characteristics of participants (i.e., age/BMI/ovarian reserve) and DHEA supplement protocol (i.e., intervention dose/duration), which makes difficulty in the integration of their findings. Importantly, the reproductive prognosis differs extremely between populations. For example, Xu et al. (9) evaluated 3391 women with POR and revealed that the live birth rate varied according to age group, which highlights the importance of covariates other than DHEA supplementation in prognosis and presents a challenge in conducting the pooled analysis. During 2021 and 2022, there was an increased number of papers with large sample size published concerning DHEA treatment in PORs. Therefore, we conducted this meta-analysis and meta-regression analysis to quantify the outcomes of PORs with DHEA treatment and explore possible factors of the heterogeneity of results, as a reference for clinical application of DHEA priming in women with POR or DOR undergoing IVF/ICSI.

## 2 Materials and methods

A PRISMA (Preferred Reporting Items for Systematic Reviews and Meta-Analyses) checklist was created and showed in the **Supplementary Materials**. The study was previously registered on PROSPERO with ID CRD42022384393.

### 2.1 Search strategy and selection criteria

We systematically performed an extensive literature search of PubMed, Web of Science, Cochrane Library, China National Knowledge Infrastructure (CNKI) from inception to Oct 2022, for all relevant articles under the following Medical Subject Headings (MeSH) terms (https://www.nlm.nih.gov/mesh/MBrowser.html) to generate subsets of studies: (i) “DHEA” or “Dehydroepiandrosterone”, (ii) “Poor ovarian response” or “POR” or “poor responder” or “diminished ovarian reserve” or “DOR”, (iii) “IVF” or “ICSI”. These subsets were combined using ‘AND’ to generate a subset of citations relevant to the research subject. Only articles reporting human participants were included. There was no language restriction of publications.

RCTs, case-controlled studies, and self-controlled studies were eligible that met the inclusion criteria: (i) participants with DOR or POR who were undergoing IVF/ICSI; (ii) patients in the study group receiving DHEA treatment, and those in the control group receiving no DHEA treatment; (iii) reported any of our outcomes of interest. The term “DOR” refers to women with advanced maternal age and/or an abnormal ovarian reserve test. There are a variety of definitions of POR among previous studies, including: (i) the initial criteria that women with advanced female age, poor ovarian response to gonadotropin stimulation, and abnormal markers of ovarian reserve; (ii) the Bologna criteria proposed in 2011 (10); and (iii) the POSEIDON criteria proposed in 2016 (11), and we did not adopt any restrictions. The primary outcomes were clinical pregnancy and live birth rate, and the secondary outcomes were the changes of AFC, AMH and bFSH, total dose of gonadotropins (Gn), stimulation days, estradiol (E2) level on the day of administering human chorionic gonadotropin (hCG), endometrial thickness, oocytes retrieved, embryos transferred and miscarriage rate. Duplicate studies and studies without full-text were excluded, as well as animal studies, conferences, and case reports.

### 2.2 Data extraction and quality assessment

All full manuscripts were reviewed by two reviewers (JZ and HJ) independently. Extraction data for each study are as follows: (i) study data (author, year of publication and country of origin); (ii) study design (sample size, blinding and allocation methods); (iii) inclusion criteria (the definitions of DOR or POR); (iv) baseline characteristics (age, BMI, bFSH and duration of infertility); (v) DHEA supplement protocol (dose and duration); (vi) stimulation protocol; (vii) outcome data. The searches and inclusion analysis were independently conducted by two researchers, JZ and HJ, and any discrepancies were resolved by discussion. They independently assessed each study’s risk of bias, and a third author (YC) resolved the disagreement. The revised Cochrane risk of bias tool for randomized trials (RoB2) (12) was used to assess the risk of bias for RCTs. The risk of bias of non-randomized studies was assessed using the risk of bias in non-randomized studies of interventions (ROBINS-I) tool (13).

### 2.3 Statistical analysis

The analysis was carried out using Stata version 17.0 (StataCorp, College Station, TX, USA). Dichotomous variate was expressed as the pooled relative risk (RR), and continuous variate was expressed as the weighted mean difference (WMD). A subgroup analysis by the type of study design was performed to evaluate the effect size and explore the source of heterogeneity. Forest plot was used to present the data graphically. Heterogeneity was evaluated with *I^2^* statistics. If the heterogeneity was not statistically significant (*I^2^* <50%), a fixed effect model using the Mantel-Haenszel method was used to analyze the outcome; if the heterogeneity was statistically significant (*I^2^* >50%), a random effect model using the DerSimonian-Laird method was used for meta-analysis. In addition, meta-regression was further planned to explore the source of high heterogeneity. The funnel plot and Egger’s test were used to evaluate publication bias. *P*<0.05 was considered statistically significant.

## 3 Results

### 3.1 Study selection and characteristics

**Figure 1:**
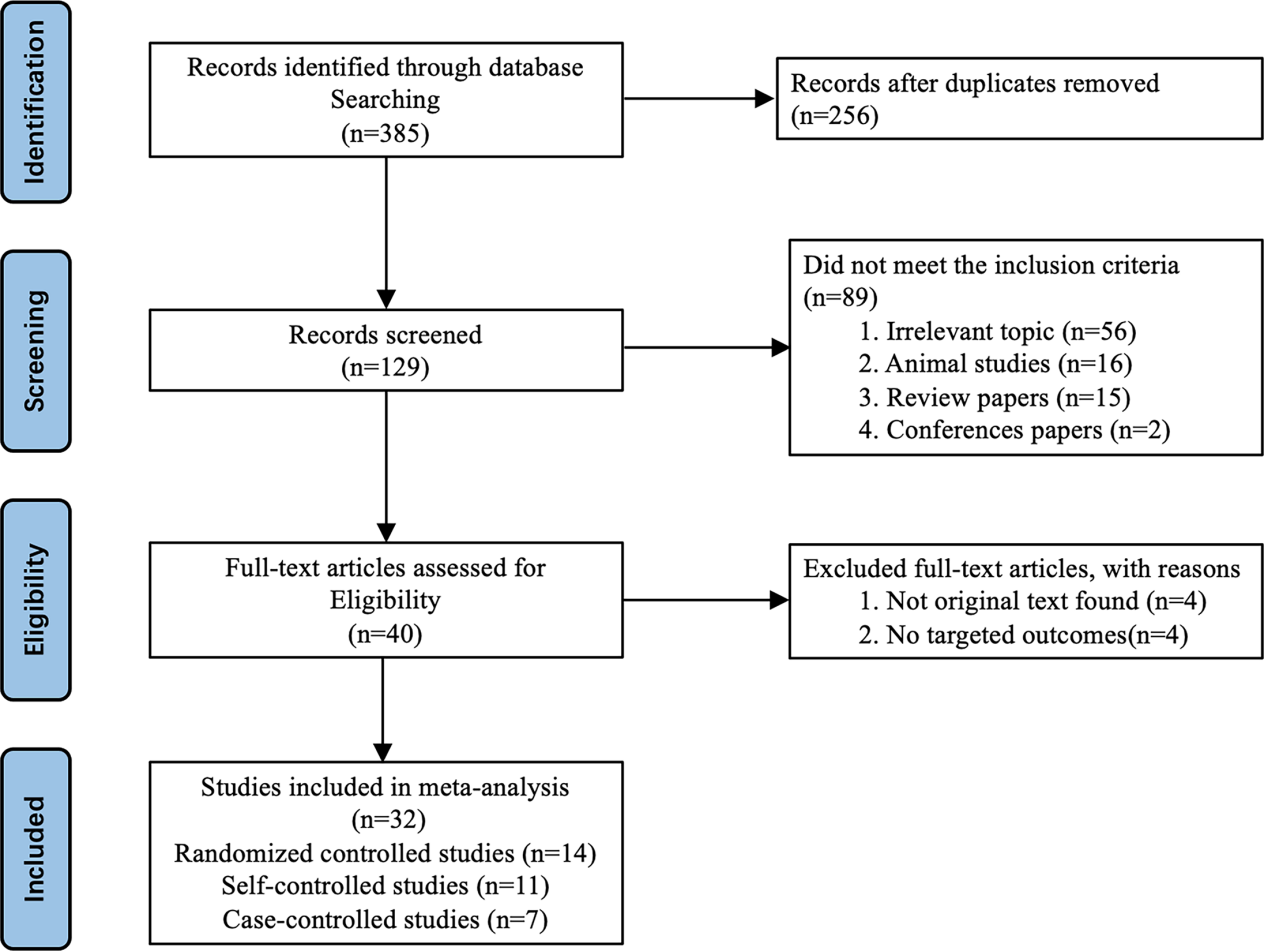
PRISMA flowchart of the study selection procedure.

The study selection process was showed in **Figure 1**. A total of 385 relevant studies were identified. After the duplicated articles were excluded, 129 studies were selected for the initial screening of titles. Then, 40 studies were in line with the experimental design by reviewing the titles and abstracts. Following the evaluation of the full manuscripts, 8 studies were excluded; finally, 32 studies were retrieved for review and inclusion in this meta-analysis. These 32 studies were published between 2006 and 2022, including 14 RCTs (14–27), 11 self-controlled studies (28–38) and 7 case-controlled studies (39–45). RCTs were assessed using the ROB2 criteria (**Figure S1**), and non-RCTs using ROBINS-I criteria (**Table S4**). The detailed characteristics of these studies were summarized in **Table S3**.

### 3.2 Primary outcomes: effect on clinical outcomes

**Figure 2:**
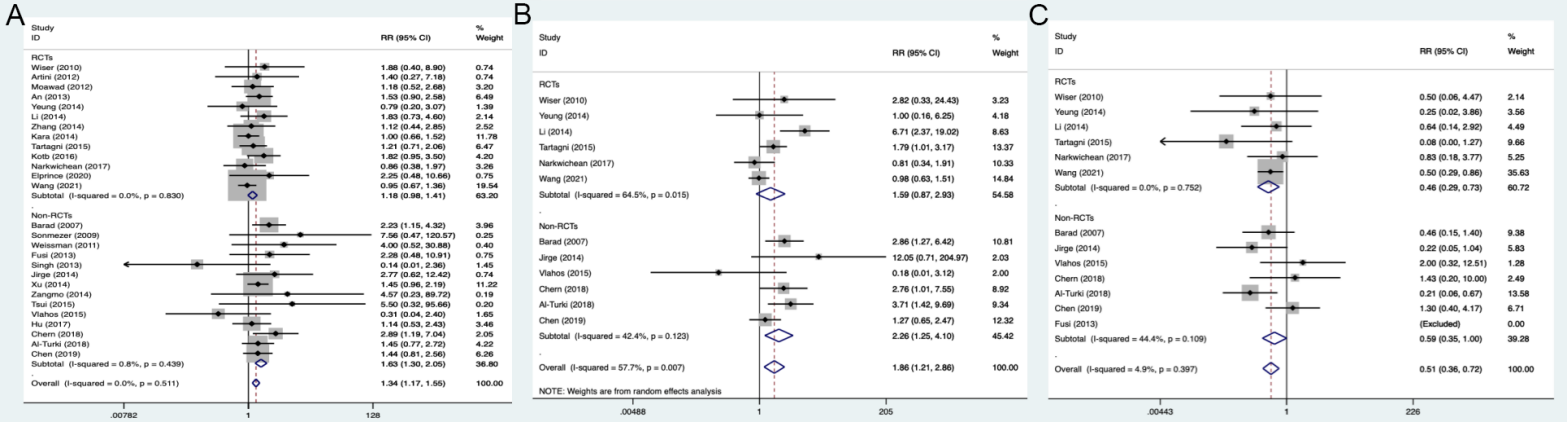
Forest plots of clinical outcomes. **(A)** Clinical pregnancy rate; **(B)** Live birth rate; **(C)** Miscarriage rate.

The clinical pregnancy rate and the live birth rate were analyzed using fixed-effect models because of low heterogeneity. For the pooled analysis of two types of studies, the DHEA supplementation groups had a higher clinical pregnancy rate (RR 1.34, 95% CI: 1.17 to 1.55, *P*<0.001) and a live birth rate (RR 1.86, 95% CI: 1.21 to 2.86, *P=*0.005) than the control groups (**Figures 2A, B**). Interestingly, the results of the RCTs showed that DHEA treatment had no relationship with the improvement of clinical pregnancy rate (RR 1.18, 95% CI: 0.98 to 1.41, *P=*0.081), which was in agreement with the live birth rate when including only RCTs (RR 1.59, 95% CI: 0.87 to 2.93, *P=*0.134) (**Figures 2A, B**).

### 3.3 Secondary outcomes

#### 3.3.1 Effect on ovarian reserve markers

**Figure 3:**
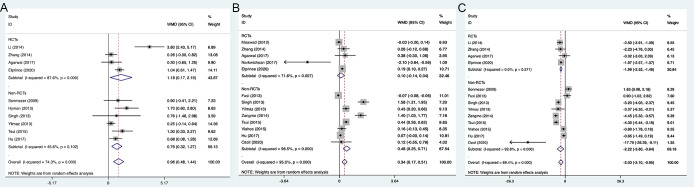
Forest plots of ovarian reserve markers. **(A)** AFC; **(B)** AMH; **(C)** FSH.

We investigated the improvement of FSH, AMH levels and AFC after DHEA treatment. The results showed that DHEA treatment significantly increased the number of AFC and decreased FSH level in subgroup analyses of only RCTs or only non-RCTs. Pooled analysis of all types of studies indicated that there was a significant increase for AMH level (WMD 0.34, 95% CI: 0.17 to 0.51, *P*<0.001) in the DHEA treatment groups than in the control groups; but for 5 RCTs, the result of pooled analysis did not display a statistical increase for AMH (WMD 0.1, 95% CI: -0.14 to 0.34, *P=*0.416) (**Figures 3A–C**).

#### 3.3.2 Effect on variables during IVF procedure

**Figure 4:**
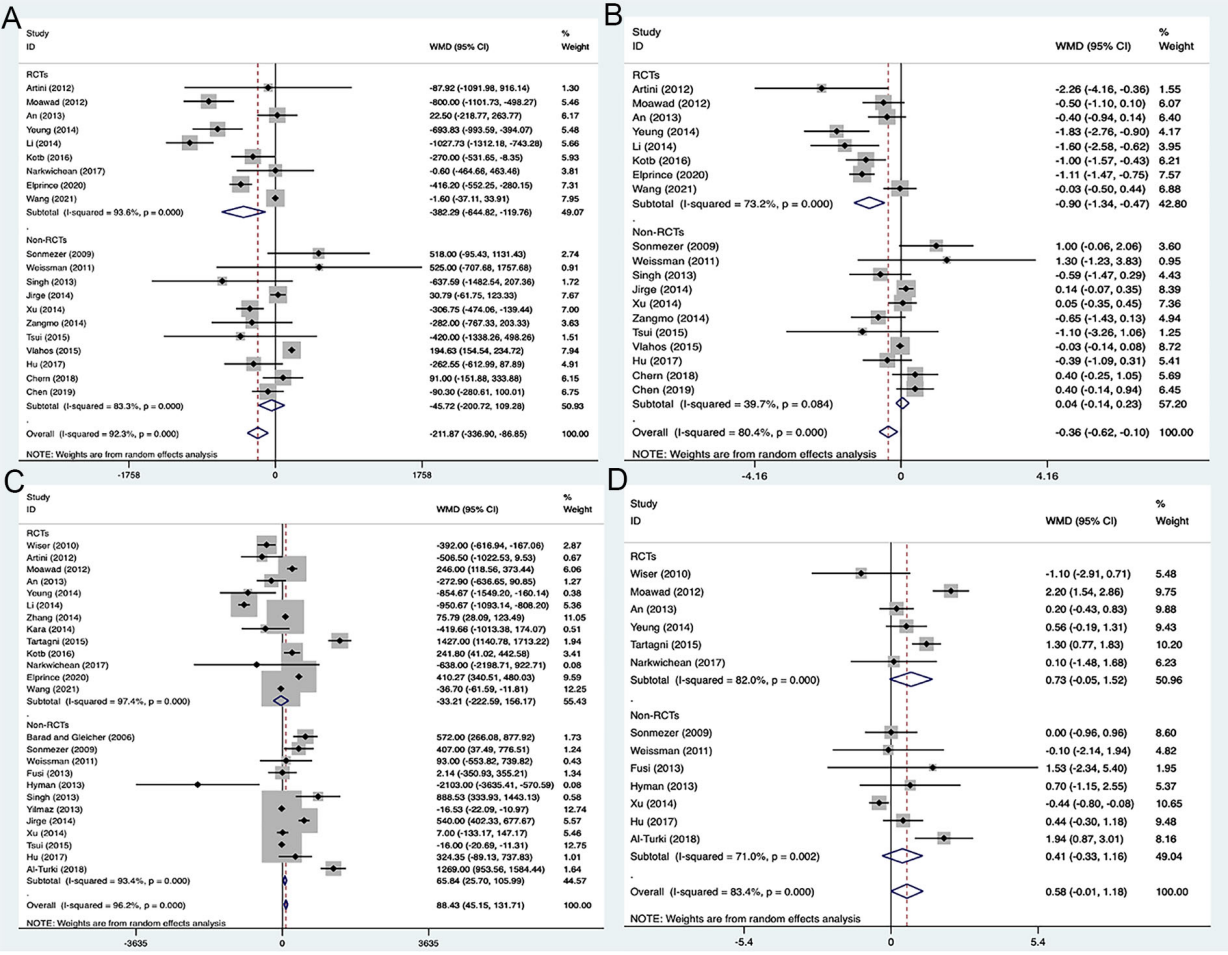
Forest plots of variables during IVF procedure. **(A)** Total doses of gonadotropin; **(B)** Days of stimulation; **(C)** E2 level on the day of hCG administration; **(D)** Endometrial thickness.

The analysis of all studies or only RCTs both showed that the needed Gn doses and stimulation days were statistically less in the DHEA treatment groups, but not in the non-RCT studies (**Figures 4A, B**). Pooled analysis of two types of studies (13 RCTs and 12 non-RCTs) indicated that DHEA treatment increased the peak E_2_ level on the day of injecting hCG (WMD 88.43, 95% CI: 45.15 to 131.71, *P*<0.001), but there was no significant difference in the only RCTs (WMD -33.21, 95% CI: -222.59 to 156.17, *P=*0.731) (**Figure 4C**). Despite the E_2_ level in the DHEA treatment group was increased in the analysis including all studies or only non-RCTs, endometrial thickness was not significantly increased (**Figures 4C, D**).

#### 3.3.3 Effect on oocytes and embryo yields

**Figure 5:**
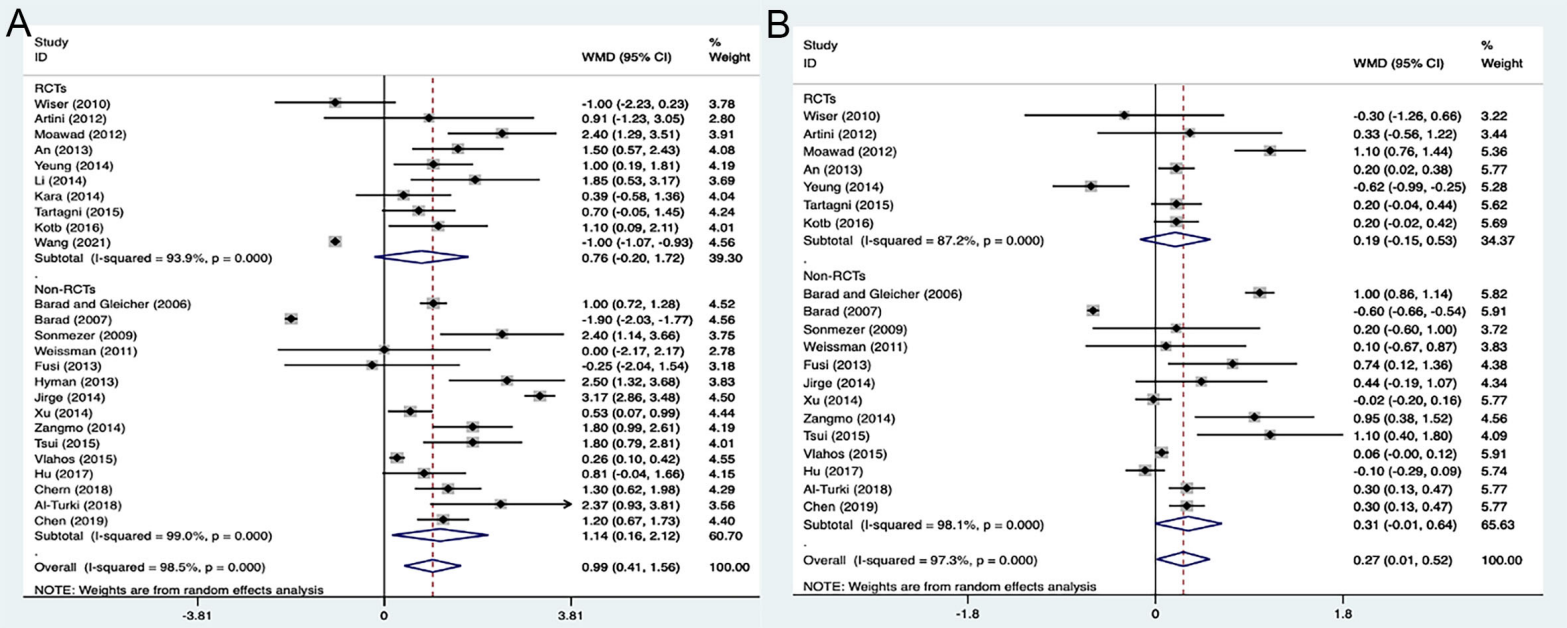
Forest plots of oocytes and embryo yields. **(A)** Number of oocytes retrieved; **(B)** Number of embryos transferred.

The pooled analysis of two types of studies showed that the DHEA treatment group had the higher numbers of retrieved oocytes (WMD 0.99, 95% CI: 0.41 to 1.56, *P=*0.001) and transferred embryos (WMD 0.27, 95% CI: 0.01 to 0.52, *P=*0.040), when compared to the control group (**Figures 5A, B**). However, no significant difference was observed in the analysis of RCTs (*P=*0.123, *P=*0.274) with high heterogeneity of 98.5% (*I^2^*) and 97.3% (*I^2^*).

#### 3.3.4 Effect on miscarriage rate

The meta-analysis revealed a low heterogeneity among the studies (*I^2^* = 4.9%). The pooled analysis was then conducted using the fixed-effects model. The results indicated that the DHEA supplementation groups had a lower miscarriage rate (RR 0.51, 95% CI: 0.36 to 0.72, *P*<0.001) than the control groups in the analysis of all studies, as well as in the subgroup analyses of only RCTs or only non-RCTs (**Figure 2C**).

### 3.4 Meta-regression analysis

**Figure 6:**
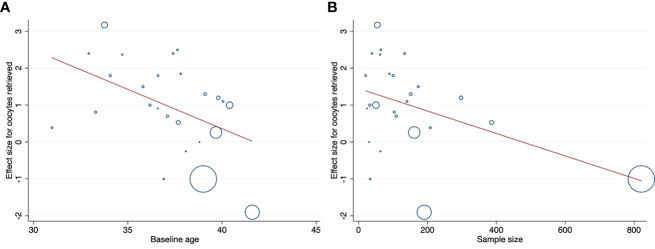
Association between study’s individual effect sizes for the number of oocytes retrieved and **(A)** baseline age of women; **(B)** sample size of trials. Plotting characters are proportional to the study weight.

To identify the source of variance in main outcomes, we conducted a series of univariate meta-regressions to examine the proportion of variance that could be explained by various individual- or study-level factors(**Table S6**). The univariable analyses showed that women with lower basal FSH had more increase in serum FSH levels (b=-0.94, 95% CI: -1.62 to -0.25, *P=*0.014), and women with higher baseline AMH levels had more increase in serum AMH levels (b=-0.60, 95% CI: -1.15 to -0.06, *P=*0.035) after DHEA supplementation. Significantly higher estradiol (E_2_) level on the day of hCG administration were found in population with higher AMH (b=222.35, 95% CI: 30.15 to 414.55, *P=*0.027) or AFC level(b=104.08, 95% CI: 2.30 to 205.85, *P=*0.046). The type of study design was a key factor of heterogeneity on stimulation days. Therefore, a subgroup analysis by the type of study design was conducted, in which including only RCTs, the use of DHEA also had a significant effect (*P*<0.001) (**Figure S3**). However, no significant effect was observed in subgroup analyses of only self-controlled (*P=*0.494) or case–controlled studies (*P=*0.960) (**Figure S3**). The retrieved oocytes were higher in the relatively younger women (b=-0.21, 95% CI: -0.39 to -0.03, *P=*0.023) (**Table 1**; **Figure 6A**) and in those studies with relatively small sample sizes (b=-0.003, 95% CI: -0.006 to -0.0003, *P=*0.032) (**Table 1**; **Figure 6B**). These two variables (baseline age and sample size) were therefore eligible for inclusion in the multivariable regression analysis, but they were not significantly associated with the number of the retrieved oocytes. For other outcomes, no statistically significant correlations were observed from the meta-regression analysis.

**Table 1:** Meta-regression analyses for the number of oocytes retrieved.

Variable	Univariable meta-regressions			Multivariable meta-regression		
	b	95%CI	*P*	b	95%CI	*P*
Individual-level						
Baseline age, y	-0.21	-0.39 to -0.03	0.023	-0.17	-0.35 to 0.001	0.052
Baseline BMI, kg/m^2^	0.07	-0.24 to 0.38	0.621			
Basal FSH, IU/L	-0.06	-0.26 to 0.15	0.556			
Basal E_2_, pg/mL	-0.002	-0.008 to 0.004	0.424			
AMH, ng/mL	-0.05	-0.60 to 0.50	0.839			
AFC	-0.08	-0.35 to 0.19	0.525			
Duration of infertility, y	0.30	-0.11 to 0.71	0.140			
Study-level						
Publication year	0.04	-0.11 to 0.20	0.580			
Study design						
RCTs	1(Ref.)	NA	NA			
Case-control	-0.18	-1.39 to 1.02	0.753			
Self-control	0.97	-0.23 to 2.18	0.109			
Sample size	-0.003	-0.006 to -0.0003	0.032	-0.002	-0.005 to 0.0002	0.071
Area, continent						
East Asia	1(Ref.)	NA	NA			
South Asia	1.59	-0.21 to 3.39	0.081			
West Asia	0.41	-0.86 to 1.67	0.509			
Europe	-0.54	-2.05 to 0.97	0.464			
Africa	0.16	-2.41 to 2.74	0.897			
America	-1.40	-3.15 to 0.35	0.111			
Duration of DHEA						
<3 months	1(Ref.)	NA	NA			
=3 months	0.61	-0.74 to 1.96	0.361			
>3 months	0.11	-1.63 to 1.86	0.895			

Ref., reference; NA, not applicable.

### 3.5 Publication bias analysis

Funnel plots and Egger’s test were used to determine the potential publication bias. Funnel plots for the outcomes of AFC (*P_Egger_* =0.132), FSH (*P_Egger_* =0.973), days of stimulation (*P_Egger_* =0.084), E_2_ level on the day of injecting hCG (*P_Egger_* =0.218), endometrial thickness (*P_Egger_* =0.529), clinical pregnancy rate (*P_Egger_* =0.106), live birth rate (*P_Egger_* =0.453) and miscarriage rate (*P_Egger_* =0.435) were partially symmetrical, demonstrating the low risk of publication bias (**Figure S3**). Funnel plots for the outcomes of AMH (*P_Egger_* =0.009), total doses of gonadotropin (*P_Egger_* =0.021), oocytes retrieved (*P_Egger_* =0.002) and oocytes transferred (*P_Egger_* =0.044) were asymmetrical, indicating publication bias (**Figure S2**).

## 4 Discussion

The present meta-analysis had a large sample size. The results for RCTs demonstrated that DHEA treatment had statistical effect on serum FSH levels, AFC, total doses of Gn, days of stimulation and miscarriage rate, and that the effects of DHEA treatment on serum AMH level, E_2_ level on the hCG day, endometrial thickness, number of oocytes retrieved and number of embryos transferred did not show the significant differences between the groups. Different populations had potentially different responses to DHEA treatment, we also conducted meta-regression analyses to detect the sources of high heterogeneity. Accordingly, DHEA treatment cannot improve the clinical pregnancy rate and live birth rate. Unlike previous meta-analysis studies, we also accepted case-controlled and self-controlled studies, which accounted for a large proportion of total trials performed. The results of these studies showed that DHEA treatment had benefits in terms of above outcomes, except for the total doses of Gn, days of stimulation and endometrial thickness.

The most direct criteria used to assess the effects of DHEA pre-treatment were clinical pregnancy and live birth rates in ART cycles. Our data did not show that DHEA supplementation significantly improved clinical pregnancy rate and live birth rate in patients with DOR/POR in a subgroup analysis of only RCTs, which is contrary to the results of most of previous meta-analysis studies (46–50). This is mainly because of the inclusion of the study reported by Wang et al. (25) in our meta-analysis, with the largest sample size, the results of this study therefore had a significant impact on our pooled effect sizes. This trial demonstrated that there were no significant differences in clinical pregnancy rate (RR 0.95; 95%CI: 0.67 to 1.35; *P=* 0.766) and live birth rate (RR 0.98; 95% CI: 0.63 to 1.51; *P=*0.911) between groups. Despite a significantly high clinical pregnancy rate in the DHEA treatment groups with low heterogeneity in non-RCTs (7 case-controlled studies and 7 self-controlled studies), the results should be interpreted with caution. On the one hand, such studies are typically thought to have a performance and detection bias towards more ideal outcomes because of lack of blinding; on the other hand, in the self-controlled studies, the effect of DHEA treatment was compared in the same patients before and after DHEA supplementation, and the clinical birth rate or live birth rate before DHEA supplementation were always low or 0%, which may lead to overestimation of the treatment effect.

Two recently published RCTs were included in our meta-analysis. One trial was conducted by Elprince et al. (27), which incorporated 50 women with serum AMH<1.1 ng/mL, serum FSH≥10 mIU/L and ≤15 mIU/L on day 3 of cycle, and low AFC≤4 on day 3 of cycle. The other trial was conducted by Wang et al. (25), including 821 women with the Bologna criteria for POR, which is the largest RCT up to now, and the sample size of which is comparable to a total of previous trials in evaluating DHEA pretreatment for POR women undergoing IVF. The results of two RCTs demonstrated that there were no significant differences in the number of retrieved oocytes, and the rates of clinical pregnancy, and the cumulative live births after DHEA treatment. Due to the large sample size, the inclusion of these two studies greatly increased the statistical power in our meta-analysis.

In subgroup analyses of only RCTs or only non-RCTs, DHEA treatment significantly increased the number of AFC and decreased FSH level, suggesting that the use of DHEA could be an effective strategy to improve the ovarian reserve in patients with POR/DOR. There was a significant higher level of the AMH in the analysis of non-RCTs, but not in RCTs; the conflicting results were mainly led by the limitations of small sample sizes of trials. The Gn doses and the stimulation days were statistically less in the DHEA treatment groups for RCTs, but not for non-RCTs; the lack of blinding in most of these studies may induce a possible bias when health care providers adjusted the dose and stimulation days. As a metabolic precursor for steroid production, DHEA supplementation might increase the E_2_ level on the day of hCG administration. Being consistent with Zhang’s findings (46), the higher peak E2 levels on the day of hCG administration and similar endometrial thickness were observed in women with DHEA treatment in the analysis of all types of studies. It’s worth noting that the E_2_ level on the day of hCG administration and endometrial thickness may be also affected by the stimulation protocols and duration of stimulation, which may lead to a heterogeneous result.

Female age, AMH, AFC, basal FSH and estradiol levels are factors that predict ovarian reserve and ovarian response (51). We found that patients with better ovarian reserve could obtain the greater improvement in ovarian reserve from DHEA supplementation, as univariate meta-regression results showed that younger women tend to have more oocytes retrieved; women with lower basal FSH had more increase in serum FSH level; women with higher baseline AMH level had more increase in serum AMH level from DHEA supplementation. Consistent with our results, the study conducted by Yeung et al. (52) showed that women with lower FSH may respond to DHEA supplementation better. It has been well accepted that women are born with all the primordial follicles and additional primordial follicles cannot be produced. Women who have less depleted ovarian reserve at the start with more primordial follicles remained for DHEA to work upon may respond better. However, further studies are needed to prove this concept. Too few studies monitored serum concentration of basal androgen in POR patients before DHEA supplementation. Patients with low DHEA-S levels, implying adrenal hypoandrogenism, could benefit more from DHEA supplementation. Chern et al. (44) showed that PORs with lower DHEA-S concentration had significantly more retrieved oocytes compared to those with higher DHEA-S concentration. Gleicher et al. (53) demonstrated that patients with secondary ovarian insufficiency induced by adrenal hypoandrogenism dramatically improved in ovarian function after DHEA supplementation. In view of those limited studies, the relationship between the basal levels of different forms of androgens and the effect size of DHEA supplementation would definitely be worth further exploration.

Univariate analyses also showed that significantly higher E_2_ level on the day of hCG administration were found in women with higher AMH or AFC level, which may result in an increase in the number of follicles in the growing pools that are susceptible to stimulation and thus secrete more follicular estrogen. The DHEA treatment groups had significantly higher number of retrieved oocytes for all types of studies. In the meta-analysis regression, we found that the number of retrieved oocytes were somewhat higher in younger women. The results also showed that the source of heterogeneity for the number of retrieved oocytes was associated with the sample sizes of trials, as overestimation of the treatment effect was more likely in trials with relatively small sample sizes. Given advances in the techniques of IVF/ICSI through years, univariate analyses of publication year were performed, which showed that there was a decrease in the effect of DHEA supplementation on the clinical pregnancy rate and live birth rate in more recent studies, but the difference was not statistically significant (*P* = 0.074 and 0.062). The reason could be that recent studies used better stimulation protocols, adopted blastocyst culture and transfer and randomized a large number of participants, which may weaken the effect of DHEA supplementation. More recent studies may better reflect practice nowadays and will be more applicable to the modern POR population than older publications.

Successful embryo implantation depends on not only high-quality embryos but also good endometrial receptivity whether for fresh embryo transfer or FET. To date, there is the limited evidence to interpret the effect of DHEA on endometrial function. Gibson et al. (54) performed *in vitro* experiments and showed that the addition of DHEA to primary human endometrial stromal fibroblasts (hESF) derived from women of advanced reproductive age increased the expressions of those decidualization markers such as IGFBP1 and PRL, and those endometrial receptivity markers such as SPP1. Gibson et al. also showed that the simultaneous addition of the AR antagonist, flutamide, reduced the expressions of both decidualization and endometrial receptivity markers. Qin et al. (55) demonstrated that DHEA could increase a receptivity-associated marker, HOXA10, and reduce production of reactive oxygen species in murine endometrial stromal fibroblasts *via* androgen receptor. Interestingly, treatment with the supraphysiological level of DHEA is not effective and decidualization is weakened (56). Additionally, in the mouse model, androgen deficiency delays embryo implantation, while excess androgen results in abnormal gene expression at the site of implantation (57). Therefore, the presence of a biphasic dose-response suggests that the therapeutic effect of DHEA depends on the careful dosing. However, more research is needed to investigate the optimal therapeutic window of DHEA treatment. Clinically, current studies intended to explore the beneficial effect of DHEA in oocyte development and/or subsequent embryo quality, which focused on only fresh ET cycles or the first chronological transfer cycle, either the fresh ET or FET cycle; its potential role in the endometrium is not explored well. To the best of our knowledge, there are no published studies on the separate FET cycles or subsequent FET cycles derived from DHEA used in stimulated cycles. It’s expected that an ongoing randomized, controlled trial (58) to evaluate whether DHEA supplementation during the luteal phase of the FET cycle improves the live birth rates will give a definitive answer, as the embryos were generated previously without DHEA exposure.

Although strong evidence from basic research indicating DHEA had a potential positive link with folliculogenesis and endometrium function, when analyzing data from only RCT studies, such a benefit was not obvious in the present meta-analysis. First, the POR population tend to have multiple factors associated with subfertility, influencing the mechanisms of implantation and subsequent pregnancy establishment. Secondly, there was a wide inconsistency in the definition of poor responders between studies. Although the Bologna criteria was proposed to standardize the definition of POR, heterogeneity still existed among POR patients based on Bologna criteria, posing many difficulties and doubts on treatment. It’s known that age is the main predictor for IVF/ICSI cycle outcome because the older age brings DOR with decreased oocyte quality. In addition, the reference range for AMH in Bologna criteria was also broad, resulting in big differences in the initial ovarian reserve of participants between studies. Therefore, the POSEIDON criteria (11) was proposed, allowing for stratification based on the heterogeneous features of POR patients, classifies the low responder women into four groups according to age, ovarian reserve, and stimulation response with the aim of determining the prognosis. To date, there has only one case-controlled study (45) investigating DHEA pretreatment in women with POR as defined by the POSEIDON criteria. This study showed women with DHEA pretreatment had significantly higher numbers of retrieved oocytes, but not a significant benefit on clinical pregnancy or live birth rates. Future studies are needed to incorporate factors outside of the traditional Bologna criteria and possibly take a more nuanced approach like that of the POSEIDON study to identify the specific population who will be benefited from DHEA before further evaluating DHEA as an intervention in standard IVF practice. Last but not least, it is possible that the duration of intervention may be insufficient to generate favorable changes in ovarian response and pregnancy outcomes following IVF. It has been suggested that the administration of DHEA for at least 3 months is needed to exhibit the maximum effect, in keeping with the approximate time interval required for the initiation of follicular recruitment (59). However, women at an advanced age or with DOR generally have the limited time left to conceive using autologous oocytes. They are more likely to indicate a demand for rapid IVF treatment. For example, the duration of intervention in the study conducted by Wang and his colleagues was designed as a flexible period (ranged from 4 to 12 weeks), and as expected, no more than half of the women were exposed to DHEA for 12 weeks, which reflects a ‘real life’ situation.

The present meta-analysis had a large sample size, and took different approaches including subgroup and meta-regression analyses, to explore the effect of DHEA on the ovaries. It also had several limitations. Firstly, most of the trials had relatively small sample sizes, which may affect the validity and reliability of our results. Secondly, combining the results of the studies has been difficult, in part due to wide variations in the baseline characteristics of populations, definitions used for DOR and POR, and differences in DHEA treatment and stimulation protocols between studies. Finally, most included studies, especially those non-RCTs, are of low to moderate quality with high risk of bias.

In conclusion, our systematic review of the randomized controlled studies suggested that DHEA treatment cannot improve clinical pregnancy rate and live birth rate in patients with DOR or POR. Based on these data, DHEA adjuvant therapy could not be recommended in those poor ovarian responders for improving IVF outcome. However, there were wide variations between trials. The controversy will also probably continue. It is required to have further large studies using more explicit selection criteria for the participants to reduce the heterogeneity so as to confirm the effects of DHEA treatment.

## Data availability statement

The original contributions presented in the study are included in the article/**Supplementary Material**. Further inquiries can be directed to the corresponding authors.

## Author contributions

JZ and YC designed the study and evaluated the data. JZ and HJ collected the information and analyzed the data. YC and JL are the doctoral advisors of JZ and HJ. FD and XM participated in the clinical and statistical analysis. JZ wrote the manuscript, YC critically revised the manuscript. All authors reviewed and approved the submitted version.

## References

[1] Keay SD Liversedge NH Mathur RS Jenkins JM . Assisted conception following poor ovarian response to gonadotrophin stimulation. Br J Obstet Gynaecol (1997) 104:521–7. doi: 10.1111/j.1471-0528.1997.tb11525.x 9166190

[2] Polyzos NP Drakopoulos P Parra J Pellicer A Santos-Ribeiro S Tournaye H . Cumulative live birth rates according to the number of oocytes retrieved after the first ovarian stimulation for in vitro fertilization/intracytoplasmic sperm injection: a multicenter multinational analysis including ∼15,000 women. Fertil Steril (2018) 110:661–70.e1. doi: 10.1016/j.fertnstert.2018.04.039 30196963

[3] Özkan ZS . Ovarian stimulation modalities in poor responders. Turk J Med Sci (2019) 49:959–62. doi: 10.3906/sag-1905-179 PMC7018357 31385487

[4] Ulug U Ben-Shlomo I Turan E Erden HF Akman MA Bahceci M . Conception rates following assisted reproduction in poor responder patients: a retrospective study in 300 consecutive cycles. Reprod BioMed Online (2003) 6:439–43. doi: 10.1016/s1472-6483(10)62164-5 12831590

[5] Burger HG . Androgen production in women. Fertil Steril (2002) 77(Suppl 4):S3–5. doi: 10.1016/s0015-0282(02)02985-0 12007895

[6] Narkwichean A Jayaprakasan K Maalouf WE Hernandez-Medrano JH Pincott-Allen C Campbell BK . Effects of dehydroepiandrosterone on *in vivo* ovine follicular development. Hum Reprod (2014) 29:146–54. doi: 10.1093/humrep/det408 24256992

[7] Walters KA . Role of androgens in normal and pathological ovarian function. Reproduction (2015) 149:R193–218. doi: 10.1530/rep-14-0517 25516989

[8] Hague WM Adams J Rodda C Brook CG de Bruyn R Grant DB . The prevalence of polycystic ovaries in patients with congenital adrenal hyperplasia and their close relatives. Clin Endocrinol (Oxf) (1990) 33:501–10. doi: 10.1111/j.1365-2265.1990.tb03887.x 2225492

[9] Xu B Chen Y Geerts D Yue J Li Z Zhu G . Cumulative live birth rates in more than 3,000 patients with poor ovarian response: a 15-year survey of final in vitro fertilization outcome. Fertil Steril (2018) 109:1051–9. doi: 10.1016/j.fertnstert.2018.02.001 29935642

[10] Ferraretti AP La Marca A Fauser BC Tarlatzis B Nargund G Gianaroli L . ESHRE consensus on the definition of 'poor response' to ovarian stimulation for *in vitro* fertilization: the Bologna criteria. Hum Reprod (2011) 26:1616–24. doi: 10.1093/humrep/der092 21505041

[11] Alviggi C Andersen CY Buehler K Conforti A De Placido G Esteves SC . A new more detailed stratification of low responders to ovarian stimulation: from a poor ovarian response to a low prognosis concept. Fertil Steril (2016) 105:1452–3. doi: 10.1016/j.fertnstert.2016.02.005 26921622

[12] Sterne JAC Savović J Page MJ Elbers RG Blencowe NS Boutron I . RoB 2: a revised tool for assessing risk of bias in randomised trials. Bmj (2019) 366:l4898. doi: 10.1136/bmj.l4898 31462531

[13] Sterne JA Hernán MA Reeves BC Savović J Berkman ND Viswanathan M . ROBINS-I: a tool for assessing risk of bias in non-randomised studies of interventions. Bmj (2016) 355:i4919. doi: 10.1136/bmj.i4919 27733354 PMC5062054

[14] Artini PG Simi G Ruggiero M Pinelli S Di Berardino OM Papini F . DHEA supplementation improves follicular microenviroment in poor responder patients. Gynecol Endocrinol (2012) 28:669–73. doi: 10.3109/09513590.2012.705386 22835219

[15] Wiser A Gonen O Ghetler Y Shavit T Berkovitz A Shulman A . Addition of dehydroepiandrosterone (DHEA) for poor-responder patients before and during IVF treatment improves the pregnancy rate: a randomized prospective study. Hum Reprod (2010) 25:2496–500. doi: 10.1093/humrep/deq220 20729538

[16] Moawad A Shaeer M . Long-term androgen priming by use of dehydroepiandrosterone (DHEA) improves IVF outcome in poor-responder patients. a randomized controlled study. Middle East Fertil Soc J (2012) 17:137–42. doi: 10.1007/s13224-016-0941-8

[17] An J Wang Y Ni YL Chai SM . Application of dehydroepiandrosterone (DHEA) supplementation in *in vitro* fertilization and embryo transfer cycles. Reprod Contracep (2013) 33:89–92. doi: 10.1007/s13224-016-0941-8

[18] Kara M Aydin T Aran T Turktekin N Ozdemir B . Does dehydroepiandrosterone supplementation really affect IVF-ICSI outcome in women with poor ovarian reserve? Eur J Obstet Gynecol Reprod Biol (2014) 173:63–5. doi: 10.1016/j.ejogrb.2013.11.008 24331115

[19] Li J Ren CE Zhang AQ Du T . Effect of dehydroepiandrosterone (DHEA) supplementation on IVF-ET of patients with diminished ovarian reserve (DOR). J Liaoning Med Univ (2014) 35:51–3. doi: 10.1136/bmj.i4919

[20] Yeung TW Chai J Li RH Lee VC Ho PC Ng EH . A randomized, controlled, pilot trial on the effect of dehydroepiandrosterone on ovarian response markers, ovarian response, and *in vitro* fertilization outcomes in poor responders. Fertil Steril (2014) 102:108–15.e1. doi: 10.1016/j.fertnstert.2014.03.044 24796766

[21] Zhang HH Xu PY Wu J Zou WW Xu XM Cao XY . Dehydroepiandrosterone improves follicular fluid bone morphogenetic protein-15 and accumulated embryo score of infertility patients with diminished ovarian reserve undergoing *in vitro* fertilization: a randomized controlled trial. J Ovarian Res (2014) 7:93. doi: 10.1186/s13048-014-0093-3 25330837 PMC4210503

[22] Tartagni M Cicinelli MV Baldini D Tartagni MV Alrasheed H DeSalvia MA . Dehydroepiandrosterone decreases the age-related decline of the *in vitro* fertilization outcome in women younger than 40 years old. Reprod Biol Endocrinol (2015) 13:18. doi: 10.1186/s12958-015-0014-3 25884390 PMC4355976

[23] Kotb MM Hassan AM AwadAllah AM . Does dehydroepiandrosterone improve pregnancy rate in women undergoing IVF/ICSI with expected poor ovarian response according to the Bologna criteria? a randomized controlled trial. Eur J Obstet Gynecol Reprod Biol (2016) 200:11–5. doi: 10.1016/j.ejogrb.2016.02.009 26963897

[24] Narkwichean A Maalouf W Baumgarten M Polanski L Raine-Fenning N Campbell B . Efficacy of dehydroepiandrosterone (DHEA) to overcome the effect of ovarian ageing (DITTO): a proof of principle double blinded randomized placebo controlled trial. Eur J Obstet Gynecol Reprod Biol (2017) 218:39–48. doi: 10.1016/j.ejogrb.2017.09.006 28934714

[25] Wang Z Yang A Bao H Wang A Deng X Xue D . Effect of dehydroepiandrosterone administration before *in vitro* fertilization on the live birth rate in poor ovarian responders according to the Bologna criteria: a randomised controlled trial. BJOG (2022) 129:1030–8. doi: 10.1111/1471-0528.17045 34882964

[26] Agarwal R Shruthi R Radhakrishnan G Singh A . Evaluation of dehydroepiandrosterone supplementation on diminished ovarian reserve: a randomized, double-blinded, placebo-controlled study. J Obstet Gynaecol India (2017) 67:137–42. doi: 10.1007/s13224-016-0941-8 PMC5371526 28405122

[27] Elprince M Kishk EA Metawie OM Albiely MM . Ovarian stimulation after dehydroepiandrosterone supplementation in poor ovarian reserve: a randomized clinical trial. Arch Gynecol Obstet (2020) 302:529–34. doi: 10.1007/s00404-020-05603-5 32451660

[28] Barad D Gleicher N . Effect of dehydroepiandrosterone on oocyte and embryo yields, embryo grade and cell number in IVF. Hum Reprod (2006) 21:2845–9. doi: 10.1093/humrep/del254 16997936

[29] Sönmezer M Ozmen B Cil AP Ozkavukçu S Taşçi T Olmuş H . Dehydroepiandrosterone supplementation improves ovarian response and cycle outcome in poor responders. Reprod BioMed Online (2009) 19:508–13. doi: 10.1016/j.rbmo.2009.06.006 19909591

[30] Weissman A Horowitz E Ravhon A Golan A Levran D . Dehydroepiandrosterone supplementation increases baseline follicular phase progesterone levels. Gynecol Endocrinol (2011) 27:1014–7. doi: 10.3109/09513590.2011.569611 21500990

[31] Fusi FM Ferrario M Bosisio C Arnoldi M Zanga L . DHEA supplementation positively affects spontaneous pregnancies in women with diminished ovarian function. Gynecol Endocrinol (2013) 29:940–3. doi: 10.3109/09513590.2013.819087 23889217

[32] Hyman JH Margalioth EJ Rabinowitz R Tsafrir A Gal M Alerhand S . DHEA supplementation may improve IVF outcome in poor responders: a proposed mechanism. Eur J Obstet Gynecol Reprod Biol (2013) 168:49–53. doi: 10.1016/j.ejogrb.2012.12.017 23312476

[33] Singh N Zangmo R Kumar S Roy KK Sharma JB Malhotra N . A prospective study on role of dehydroepiandrosterone (DHEA) on improving the ovarian reserve markers in infertile patients with poor ovarian reserve. Gynecol Endocrinol (2013) 29:989–92. doi: 10.3109/09513590.2013.824957 24004296

[34] Yilmaz N Uygur D Inal H Gorkem U Cicek N Mollamahmutoglu L . Dehydroepiandrosterone supplementation improves predictive markers for diminished ovarian reserve: serum AMH, inhibin b and antral follicle count. Eur J Obstet Gynecol Reprod Biol (2013) 169:257–60. doi: 10.1016/j.ejogrb.2013.04.003 23664458

[35] Jirge PR Chougule SM Gavali VG Bhomkar DA . Impact of dehydroepiandrosterone on clinical outcome in poor responders: a pilot study in women undergoing *in vitro* fertilization, using bologna criteria. J Hum Reprod Sci (2014) 7:175–80. doi: 10.4103/0974-1208.142477 PMC4229792 25395742

[36] Zangmo R Singh N Kumar S Vanamail P Tiwari A . Role of dehydroepiandrosterone in improving oocyte and embryo quality in IVF cycles. Reprod BioMed Online (2014) 28:743–7. doi: 10.1016/j.rbmo.2014.01.019 24745834

[37] Tsui KH Lin LT Chang R Huang BS Cheng JT Wang PH . Effects of dehydroepiandrosterone supplementation on women with poor ovarian response: a preliminary report and review. Taiwan J Obstet Gynecol (2015) 54:131–6. doi: 10.1016/j.tjog.2014.07.007 25951716

[38] Ozcil MD . Dehydroepiandrosterone supplementation improves ovarian reserve and pregnancy rates in poor responders. Eur Rev Med Pharmacol Sci (2020) 24:9104–11. doi: 10.26355/eurrev_202009_22856 32965000

[39] Barad D Brill H Gleicher N . Update on the use of dehydroepiandrosterone supplementation among women with diminished ovarian function. J Assist Reprod Genet (2007) 24:629–34. doi: 10.1007/s10815-007-9178-x PMC3454995 18071895

[40] Xu B Li Z Yue J Jin L Li Y Ai J . Effect of dehydroepiandrosterone administration in patients with poor ovarian response according to the Bologna criteria. PloS One (2014) 9:e99858. doi: 10.1371/journal.pone.0099858 24932478 PMC4059703

[41] Vlahos N Papalouka M Triantafyllidou O Vlachos A Vakas P Grimbizis G . Dehydroepiandrosterone administration before IVF in poor responders: a prospective cohort study. Reprod BioMed Online (2015) 30:191–6. doi: 10.1016/j.rbmo.2014.10.005 25498594

[42] Hu Q Hong L Nie M Wang Q Fang Y Dai Y . The effect of dehydroepiandrosterone supplementation on ovarian response is associated with androgen receptor in diminished ovarian reserve women. J Ovarian Res (2017) 10:32. doi: 10.1186/s13048-017-0326-3 28472976 PMC5418866

[43] Al-Turki HA . Dehydroepiandrosterone supplementation in women undergoing assisted reproductive technology with poor ovarian response. a prospective case-control study. J Int Med Res (2018) 46:143–9. doi: 10.1177/0300060517720005 PMC6011324 28758852

[44] Chern CU Tsui KH Vitale SG Chen SN Wang PH Cianci A . Dehydroepiandrosterone (DHEA) supplementation improves *in vitro* fertilization outcomes of poor ovarian responders, especially in women with low serum concentration of DHEA-s: a retrospective cohort study. Reprod Biol Endocrinol (2018) 16:90. doi: 10.1186/s12958-018-0409-z 30223902 PMC6142344

[45] Chen SN Tsui KH Wang PH Chern CU Wen ZH Lin LT . Dehydroepiandrosterone supplementation improves the outcomes of *in vitro* fertilization cycles in older patients with diminished ovarian reserve. Front Endocrinol (Lausanne) (2019) 10:800. doi: 10.3389/fendo.2019.00800 31803144 PMC6873389

[46] Zhang M Niu W Wang Y Xu J Bao X Wang L . Dehydroepiandrosterone treatment in women with poor ovarian response undergoing IVF or ICSI: a systematic review and meta-analysis. J Assist Reprod Genet (2016) 33:981–91. doi: 10.1007/s10815-016-0713-5 PMC4974220 27094195

[47] Li J Yuan H Chen Y Wu H Wu H Li L . A meta-analysis of dehydroepiandrosterone supplementation among women with diminished ovarian reserve undergoing *in vitro* fertilization or intracytoplasmic sperm injection. Int J Gynaecol Obstet (2015) 131:240–5. doi: 10.1016/j.ijgo.2015.06.028 26421833

[48] Liu Y Hu L Fan L Wang F . Efficacy of dehydroepiandrosterone (DHEA) supplementation for *in vitro* fertilization and embryo transfer cycles: a systematic review and meta-analysis. Gynecol Endocrinol (2018) 34:178–83. doi: 10.1080/09513590.2017.1391202 29073790

[49] Schwarze JE Canales J Crosby J Ortega-Hrepich C Villa S Pommer R . DHEA use to improve likelihood of IVF/ICSI success in patients with diminished ovarian reserve: a systematic review and meta-analysis. JBRA Assist Reprod (2018) 22:369–74. doi: 10.5935/1518-0557.20180046 PMC6210617 30125071

[50] Xu L Hu C Liu Q Li Y . The effect of dehydroepiandrosterone (DHEA) supplementation on IVF or ICSI: a meta-analysis of randomized controlled trials. Geburtshilfe Frauenheilkd (2019) 79:705–12. doi: 10.1055/a-0882-3791 PMC6620181 31303658

[51] Keane K Cruzat VF Wagle S Chaudhary N Newsholme P Yovich J . Specific ranges of anti-mullerian hormone and antral follicle count correlate to provide a prognostic indicator for IVF outcome. Reprod Biol (2017) 17:51–9. doi: 10.1016/j.repbio.2016.12.002 28132758

[52] Yeung TW Li RH Lee VC Ho PC Ng EH . A randomized double-blinded placebo-controlled trial on the effect of dehydroepiandrosterone for 16 weeks on ovarian response markers in women with primary ovarian insufficiency. J Clin Endocrinol Metab (2013) 98:380–8. doi: 10.1210/jc.2012-3071 23144466

[53] Gleicher N Kushnir VA Weghofer A Barad DH . The importance of adrenal hypoandrogenism in infertile women with low functional ovarian reserve: a case study of associated adrenal insufficiency. Reprod Biol Endocrinol (2016) 14:23. doi: 10.1186/s12958-016-0158-9 27112552 PMC4845439

[54] Gibson DA Simitsidellis I Kelepouri O Critchley HOD Saunders PTK . Dehydroepiandrosterone enhances decidualization in women of advanced reproductive age. Fertil Steril (2018) 109:728–34.e2. doi: 10.1016/j.fertnstert.2017.12.024 29397924 PMC5908781

[55] Qin A Qin J Jin Y Xie W Fan L Jiang L . DHEA improves the antioxidant capacity of endometrial stromal cells and improves endometrium receptivity *via* androgen receptor. Eur J Obstet Gynecol Reprod Biol (2016) 198:120–6. doi: 10.1016/j.ejogrb.2016.01.016 26835588

[56] Young SL . Androgens and endometrium: new lessons from the corpus luteum *via* the adrenal cortex? Fertil Steril (2018) 109:623–4. doi: 10.1016/j.fertnstert.2018.01.027 29653715

[57] Diao HL Su RW Tan HN Li SJ Lei W Deng WB . Effects of androgen on embryo implantation in the mouse delayed-implantation model. Fertil Steril (2008) 90:1376–83. doi: 10.1016/j.fertnstert.2007.07.1341 18053999

[58] Noushin MA Sahu A Singh S Singh S Jayaprakasan K Basheer R . Dehydroepiandrosterone (DHEA) role in enhancement and maintenance of implantation (DREAM): randomised double-blind placebo-controlled trial-study protocol. BMJ Open (2021) 11:e054251. doi: 10.1136/bmjopen-2021-054251 PMC8552157 34706964

[59] Gougeon A . Dynamics of follicular growth in the human: a model from preliminary results. Hum Reprod (1986) 1:81–7. doi: 10.1093/oxfordjournals.humrep.a136365 3558758

