# Supplementary material for: Efficacy of dehydroepiandrosterone priming in women with poor ovarian response undergoing IVF/ICSI: a meta-analysis
Source: Front Endocrinol (Lausanne). 2023 Jun 9;14:1156280. doi: 10.3389/fendo.2023.1156280 (PMC10288189; doi:10.3389/fendo.2023.1156280)
Supplement: Supplementary file 1 [file DataSheet_1.pdf]

Supplementary Materials

Table legends

Table S1. PRISMA 2020 checklist.

Table S2. Search strategy.

Table S3. Characteristics of the included studies.

Table S4. Results of quality assessment using ROBINS-I for non-RCTs.

Table S5. Meta-analyses of patients treated with DHEA or not.

Table S6. Univariable meta-regression analyses for outcomes with significant difference.

Figure legends

Figure S1. Results of quality assessment using ROB2 for RCTs.

Figure S2. Meta-analysis of studies of DHEA supplementation for outcomes of **(A)** AFC; **(B)** AMH; **(C)** FSH; **(D)** Total doses of gonadotropin; **(E)** Days of stimulation; **(F)** E_2 level on the day of hCG administration; **(G)** Endometrial thickness; **(H)** Number of oocytes retrieved; **(I)** Number of oocytes transferred; **(J)** Miscarriage rate.

Figure S3. The subgroup meta-analysis by type of study design for the outcome of days of stimulation in poor responders undergoing IVF treatment.

Table S1. PRISMA 2020 checklist.

Section and Topic	Item #	Checklist item	Location where item is reported
**TITLE**			
Title	1	Identify the report as a systematic review.	1
**ABSTRACT**			
Abstract	2	See the PRISMA 2020 for Abstracts checklist.	1
**INTRODUCTION**			
Rationale	3	Describe the rationale for the review in the context of existing knowledge.	2
Objectives	4	Provide an explicit statement of the objective(s) or question(s) the review addresses.	3
**METHODS**			
Eligibility criteria	5	Specify the inclusion and exclusion criteria for the review and how studies were grouped for the syntheses.	3
Information sources	6	Specify all databases, registers, websites, organisations, reference lists and other sources searched or consulted to identify studies. Specify the date when each source was last searched or consulted.	3
Search strategy	7	Present the full search strategies for all databases, registers and websites, including any filters and limits used.	3, Table S2
Selection process	8	Specify the methods used to decide whether a study met the inclusion criteria of the review, including how many reviewers screened each record and each report retrieved, whether they worked independently, and if applicable, details of automation tools used in the process.	3
Data collection process	9	Specify the methods used to collect data from reports, including how many reviewers collected data from each report, whether they worked independently, any processes for obtaining or confirming data from study investigators, and if applicable, details of automation tools used in the process.	3
Data items	10a	List and define all outcomes for which data were sought. Specify whether all results that were compatible with each outcome domain in each study were sought (e.g. for all measures, time points, analyses), and if not, the methods used to decide which results to collect.	3
	10b	List and define all other variables for which data were sought (e.g. participant and intervention characteristics, funding sources). Describe any assumptions made about any missing or unclear information.	3
Study risk of bias assessment	11	Specify the methods used to assess risk of bias in the included studies, including details of the tool(s) used, how many reviewers assessed each study and whether they worked independently, and if applicable, details of automation tools used in the process.	4
Effect measures	12	Specify for each outcome the effect measure(s) (e.g. risk ratio, mean difference) used in the synthesis or presentation of results.	4
Synthesis	13a	Describe the processes used to decide which studies were eligible for each synthesis (e.g. tabulating the study	3

Section and Topic	Item #	Checklist item	Location where item is reported
methods		intervention characteristics and comparing against the planned groups for each synthesis (item #5)).	
	13b	Describe any methods required to prepare the data for presentation or synthesis, such as handling of missing summary statistics, or data conversions.	3
	13c	Describe any methods used to tabulate or visually display results of individual studies and syntheses.	3
	13d	Describe any methods used to synthesize results and provide a rationale for the choice(s). If meta-analysis was performed, describe the model(s), method(s) to identify the presence and extent of statistical heterogeneity, and software package(s) used.	4
	13e	Describe any methods used to explore possible causes of heterogeneity among study results (e.g. subgroup analysis, meta-regression).	4
	13f	Describe any sensitivity analyses conducted to assess robustness of the synthesized results.	4
Reporting bias assessment	14	Describe any methods used to assess risk of bias due to missing results in a synthesis (arising from reporting biases).	4
Certainty assessment	15	Describe any methods used to assess certainty (or confidence) in the body of evidence for an outcome.	4
**RESULTS**			
Study selection	16a	Describe the results of the search and selection process, from the number of records identified in the search to the number of studies included in the review, ideally using a flow diagram.	4, Figure 1
	16b	Cite studies that might appear to meet the inclusion criteria, but which were excluded, and explain why they were excluded.	4
Study characteristics	17	Cite each included study and present its characteristics.	4, Table S3
Risk of bias in studies	18	Present assessments of risk of bias for each included study.	Table S4, Figure S1
Results of individual studies	19	For all outcomes, present, for each study: (a) summary statistics for each group (where appropriate) and (b) an effect estimate and its precision (e.g. confidence/credible interval), ideally using structured tables or plots.	Table S3
Results of syntheses	20a	For each synthesis, briefly summarise the characteristics and risk of bias among contributing studies.	4-5
	20b	Present results of all statistical syntheses conducted. If meta-analysis was done, present for each the summary estimate and its precision (e.g. confidence/credible interval) and measures of statistical heterogeneity. If comparing groups, describe the direction of the effect.	4-5
	20c	Present results of all investigations of possible causes of heterogeneity among study results.	4-5
	20d	Present results of all sensitivity analyses conducted to assess the robustness of the synthesized results.	4-5

Section and Topic	Item #	Checklist item	Location where item is reported
Reporting biases	21	Present assessments of risk of bias due to missing results (arising from reporting biases) for each synthesis assessed.	4-5
Certainty of evidence	22	Present assessments of certainty (or confidence) in the body of evidence for each outcome assessed.	4-5
**DISCUSSION**			
Discussion	23a	Provide a general interpretation of the results in the context of other evidence.	5
	23b	Discuss any limitations of the evidence included in the review.	7
	23c	Discuss any limitations of the review processes used.	8
	23d	Discuss implications of the results for practice, policy, and future research.	7
**OTHER INFORMATION**			
Registration and protocol	24a	Provide registration information for the review, including register name and registration number, or state that the review was not registered.	3
	24b	Indicate where the review protocol can be accessed, or state that a protocol was not prepared.	3
	24c	Describe and explain any amendments to information provided at registration or in the protocol.	7
Support	25	Describe sources of financial or non-financial support for the review, and the role of the funders or sponsors in the review.	8
Competing interests	26	Declare any competing interests of review authors.	8
Availability of data, code and other materials	27	Report which of the following are publicly available and where they can be found: template data collection forms; data extracted from included studies; data used for all analyses; analytic code; any other materials used in the review.	8

Table S2. Search strategy.

**Search strategy of Pubmed**
("dehydroepiandrosterone"[MeSH Terms] OR "dehydroepiandrosterone" OR "dhea") AND ((("poverty"[MeSH Terms] OR "poverty" OR "poor") AND ("ovarian" OR "ovarians") AND ("response" OR "responses" OR "responsive" OR "responsiveness" OR "responsivenesses" OR "responsives" OR "responsivities" OR "responsivity")) OR ("pathol oncol res"[Journal] OR "por") OR (("poverty"[MeSH Terms] OR "poverty" OR "poor") AND ("respond" OR "respondant" OR "respondants" OR "responded" OR "respondent s" OR "responder" OR "responders" OR "responding" OR "respondings" OR "responds" OR "surveys and questionnaires"[MeSH Terms] OR ("surveys" AND "questionnaires") OR "surveys and questionnaires" OR "respondent" OR "respondents")) OR (("diminish" OR "diminished" OR "diminishes" OR "diminishing" OR "diminishment" OR "diminishments") AND ("ovarian reserve"[MeSH Terms] OR ("ovarian" AND "reserve") OR "ovarian reserve")) OR "DOR") AND ("j in vitro fert embryo transf"[Journal] OR "ivf" OR ("in vitro fertilisation" OR "fertilization in vitro"[MeSH Terms] OR ("fertilization" AND "vitro") OR "fertilization in vitro" OR ("vitro" AND "fertilization") OR "in vitro fertilization") OR ("sperm" AND "injections" AND "intracytoplasmic") OR "intracytoplasmic sperm injections" OR "icsi") OR ("sperm injections, intracytoplasmic"[MeSH Terms] OR ("sperm" AND "injections"

AND "intracytoplasmic") OR "intracytoplasmic sperm injections" OR ("intracytoplasmic" AND "sperm" AND "injection") OR "intracytoplasmic sperm injection") OR ("reproductive techniques, assisted"[MeSH Terms] OR ("reproductive" AND "techniques" AND "assisted") OR "assisted reproductive techniques" OR ("assisted" AND "reproductive" AND "technique") OR "assisted reproductive technique")
Note: Search strategies of other databases were modified accordingly.

Table S3. Characteristics of the included studies.

Study	Country	Design	Inclusion criteria	Study group/post-DHEA				Control group/pre-DHEA				Intervention		Stimulation protocol	Outcomes
				n	Age	BMI	bFSH	n	Age	BMI	bFSH	Dose	Duration		
Wiser et al. (2010)(1)	Israel	RCT	POR: <5 oocytes retrieved or previous cycle cancelation	17	36.9±4.7	26.1±5.5	9.63±3.23	16	37.8±4.6	25.7±4.6	9.82±2.97	75 mg/d	> 6-18 weeks	Long GnRH agonist	f-l
Artini et al. (2012)(2)	italy	RCT	POR: Bologna criteria	12	36.58±3.32	21.7±3.1	5.78±1.45	12	37±4.61	21.6±3.1	6.68±1.79	25 mg tid	3 months	GnRH antagonist	d-f, h-j
Moawad et al. (2012)(3)	United Arab Emirates	RCT	POR: <5 oocytes retrieved or previous cycle cancelation; and AMH<1.7 ug/L	67	37.4±6.4	29.1±3.8	NA	66	37.1±7.2	28.7±4.1	NA	25 mg tid	>12 weeks	Short GnRH agonist	c-j
An et al. (2013)(4)	China	RCT	POR: Bologna criteria	81	35.8±4.1	NA	9.3±2.6	92	35.9±2.9	NA	10±2	75 mg/d	12 weeks	Long agonist/ antagonist protocol	d-j
Yeung et al. (2014)(5)	China	RCT	POR: age≤40 years, subfertility >1 year, and AFC <5	16	36.17±0.85	21.63±0.96	11.03±1.75	16	36.8±0.85	21.23±1.13	13.72±1.55	25 mg tid	>12 weeks	GnRH antagonist protocol	d-l
Li et al. (2014)(6)	China	RCT	POR: age ≥35 or occytes retrieved <5; and AFC<5 or bFSH>10IU/L.	42	37.8±4.6	NA	10.35±3.27	47	36.9±4.7	NA	9.93±3.51	75 mg/d	12 weeks	Not reported in details	a, d-f, h, j-l
Zhang et al. (2014)(7)	China	RCT	DOR: ≥ 10 IU/L FSH or FSH/LH > 3; AFC<5; and <5 oocytes retrieved or previous cycle cancelation	42	37.17±5.22	22.13±2.92	11.68±6.62	42	37.43±4.33	22.46±2.42	10.81±2.41	75 mg/d	12 weeks	Not reported	a-c, f, j
Kara et al. (2014)(8)	Turkey	RCT	DOR: AMH <1 ng/ml or FSH >15 IU/L; and AFC<4	104	30.97±5.76	NA	16.31±0.91	104	31.15±5.58	NA	17.13±0.98	25 mg tid	12 weeks	Microdose flare	f, h, j
Tartagni et al. (2015)(9)	Italy	RCT	Age-related poor responses: 36 to 40 years and normal ovarian reserve	53	37.1±2.2	22.1±1.6	6.5±1.3	56	37.4±2.9	22.5±1.4	6.38±1.3	75 mg/d	8 weeks	Long agonist/ antagonist GnRH	f-l
Kotb et al. (2016)(10)	Egypt	RCT	POR: Bologna criteria	70	40.05±3.1	25.6±3.4	12.1±0.9	70	39.7±0.5	25.1±3.4	11.9±0.8	25 mg tid	12 weeks	GnRH antagonist	d-f, h-j
Agarwal et al. (2017)(11)	India	RCT	DOR: elevated age-specific FSH or AMH<1.05 ng/ml or AFC≤4	40	33.1±4.29	25.01±3.31	10.68±6.49	40	32.3±4.07	25.04±3.77	12.69±8.01	75 mg/d	12 weeks	Not reported	a-c
Narkwichean et al. (2017)(12)	United Kingdom	RCT	DOR: AFC<10 and/or AMH<5 pmol/l	27	36.8±3.9	24.5±4.7	NA	25	35.2±5.3	23.7±3.3	NA	75 mg/d	>12 weeks	GnRH agonist	c-h, j-l
Elprince et al. (2020)(13)	Egypt	RCT	POR: AMH < 1.1 ng/mL, bFSH ≥ 10IU/L and ≤ 15 IU/L, and AFC ≤ 4	25	36.52±2.26	23.08±1.47	12.16±0.94	25	36.28±2.53	22.56±1.8	11.94±1.11	75 mg/d	2 Continuous cycles	Not reported	a-f, j

Wang et al. (2021)(14)	China	RCT	POR: Bologna criteria	410	39±4.64	23.83±3.16	9.34±0.91	411	39.53±4.39	24.01±3.07	9.22±0.91	25 mg tid	4-12weeks	Short GnRH agonist	d-f, h, j-l
Barad and Gleicher et al. (2006)(15)	United States of America	Self-controlled	DOR: < 4 oocytes retrieved, FSH >10 IU/l or E2 >75 pg/ml and poor embryo quality	25	40.4±0.8	NA	NA	25	39.9±0.8	NA	NA	75 mg/d	17.6±2.13 weeks	Norethindrone acetate tablets	f, h-i
Barad et al. (2007)(15, 16)	United States of America	Case-controlled	POA: bFSH <12 IU/l, but exceeding the 95% CI of the mean value for the patient's age group. DOR: bFSH ≥12 IU/l, and/ or a baseline estradiol level ≥ 75 pg/ml	89	41.6±0.4	NA	16±1.2	101	40±0.4	NA	13.6±1	75 mg/d	3.8 ±0.3 months	Microdose agonist flare	h-l
Sonmezer et al. (2009)(17)	Turkey	Self-controlled	POR: cycle cancellation or <4 oocytes retrieved	19	32.92±3.76	25.04±3.99	9.72±2.19	19	32.92±3.76	25.04±3.99	9.72±2.19	75 mg/d	90-180 d	GnRH antagonist	a, b, d-j
Weissman et al. (2011)(18)	Israle	Self-controlled	Poor response: ≥2 items: < 5 oocytes retrieved, ≤3 follicles of 16 mm, or E2 <500 pg/ml	15	38.8±3.4	NA	13.3±4.7	15	38.8±3.4	NA	13.3±4.7	75 mg/d	~3 months	GnRH analog	d-j
Fusi et al. (2013)(19)	Italy	Self-controlled	DOR: AFC < 4, FSH > 10 IU/ml, AMH < 1 ng/ml	39	38.07±2.9	NA	9.67±3.9	24	38.07±2.9	NA	9.67±3.9	75 mg/d	> 3 months	long GnRH agonist/ antagonist	b, c, f-j, l
Hyman et al. (2013)(20)	Israel	Self-controlled	Poor response: ≤4 oocytes retrieved	32	37.63±5.63	NA	9.2±9	32	37.63±5.63	NA	9.2±9	75 mg/d	>3 months	GnRH antagonist	a, f-h
Singh et al. (2013)(21)	India	Self-controlled	Poor ovarian response in the previous IVF cycle(s)	17	<35	NA	NA	17	<35	NA	NA	75 mg/d	4 months	Long agonist/ antagonist	a-f, j
Yilmaz et al. (2013)(22)	Turkey	Self-controlled	DOR: AFC <5 or AMH <1.1 ng/ml, and a previous poor ovarian response	41	33.78±3.11	24.93±2.52	NA	41	33.78±3.11	24.93±2.52	NA	75 mg/d	> 6 weeks	GnRH antagonist	a-c, f
Jirge et al. (2014)(23)	India	Self-controlled	POR: Bologna criteria	25	33.76±5.13	24.19±4.94	13.8±1.69	29	33.76±5.13	24.19±4.94	13.8±1.69	90 mg/d	> 2 months	GnRH antagonist	d-f, h-l
Xu et al. (2014)(24)	China	Case-controlled	POR: Bologna criteria	189	37.67±4.67	21.77±5.9	12.34±3.79	197	38.1±4.22	21.4±5.1	11.84±3.64	75 mg/d	90 d	GnRH antagonist	d-j
Zangmo et al. (2014)(25)	India	Self-controlled	Poor response: <42 years, <5 oocytes and bFSH 10–20 IU/l	50	34.06±4	24.14±2.17	13.05±1.05	50	34.06±4	24.14±2.17	13.05±1.05	75 mg/d	4 months	GnRH agonist/ antagonist	b-e, h-j
Tsui et al. (2015)(26)	China	Self-controlled	POR: Bologna criteria	10	36.6±4.2	21.4±2.5	14.4±1.7	10	36.6±4.2	21.4±2.5	14.4±1.7	90 mg/d	12.2 weeks	GnRH antagonist	A-f, h-j
Vlahos et al. (2015)(27)	Greece	Case-controlled	Poor response: ≥2 items: >40 years, bFSH >9.5 IU/l, AMH< 2 ng/ml, < 3 oocytes	48	39.67±0.54	22.3±0.6	13.19±0.33	113	39.07±0.34	23.7±0.8	12.46±0.22	50 mg/d	> 3 months	GnRH antagonist	b-e, h-l

			retrieved, previous cycle cancelation												
Hu et al. (2017)(28)	China	Case-controlled	DOR: ≥2 items: FSH 10-25 IU/L, E_2 >80 pg/ml, AMH <0.5-1.1 ng/ml, AFC≤5	53	33.28±3.13	22.32±2.44	10.57±1.91	50	34.16±3.27	23.2±4.41	10.45±2.27	75 mg/d	8 weeks	GnRH antagonist	a-j
Chern et al. (2018)(29)	China	Case-controlled	POR: Bologna criteria	67	39.1±3.3	21.9±2.9	7.1±4.2	84	39.8±3.7	21.8±3.8	6.3±4.6	90 mg/d	3 months	GnRH antagonist	d, e, h, j-l
Al-Turki et al. (2018)(30)	Saudi Arabia	Case-controlled	POR: Bologna criteria	34	34.7±4.37	21.7±1.4	11.25±2.62	28	33.9±5.1	22.2±1.1	10.96±1.3	50 mg/d	3 months	GnRH antagonist	f-l
Chen et al. (2019)(31)	China	Case-controlled	DOR: POSEIDON group 4 criteria	159	39.8±2.5	22.3±3.1	7.3±4.5	138	40.2±2.9	23±3.7	6.3±4.1	30 mg tid	3 months	GnRH antagonist	d, e, h-l
Ozcil et al. (2020)(32)	Turkey	Self-controlled	POI: 4-month period of amenorrhea, bFSH > 40 IU/L and a decrease in sex steroids in women<40 years. POR: Bologna criteria	34	35.8±7.6	NA	NA	34	35.8±7.6	NA	NA	50 mg/d	5 months	Not reported	b, c

Abbreviations: DHEA, dehydroepiandrosterone; RCT, randomized controlled trial; AMH, anti-Müllerian hormone; bFSH, basal follicle-stimulating hormone; AFC, antral follicle count; GnRH, gonadotropin-releasing hormone; DOR, diminished ovarian reserve; POR, poor ovarian response.

a. AFC; b. AMH; c. FSH; d. Total doses of Gn; e. Days of stimulation; f. E2 level on the day of hCG; g. Endometrial thickness; h. Number of oocytes retrieved; i. Number of embryos transferred; j. Clinical pregnancy rate of IVF; k. Live birth rate; l. Miscarriage rate

Table S4. Results of quality assessment using ROBINS-I for non-RCTs.

Study	Bias due to Confounding	Bias in Selection of Participants	Bias in Classification of Interventions	Bias due to Deviations from Intended Interventions	Bias due to Missing Data	Base in Measurement of Outcomes	Bias in Selection of the Reported Results	Overall
Barad and Gleicher et al. (2006)	Moderate	Moderate	Low	Moderate	Low	Moderate	Serious	Serious
Barad et al. (2007)	Moderate	Moderate	Low	Low	Low	Moderate	Moderate	Moderate
Sonmezer et al. (2009)	Low	Moderate	Low	Low	Low	Moderate	Moderate	Moderate
Weissman et al. (2011)	Moderate	Moderate	Low	Moderate	Low	Moderate	Moderate	Moderate
Fusi et al. (2013)	Low	Low	Low	Moderate	Low	Moderate	Moderate	Moderate
Hyman et al. (2013)	Moderate	Moderate	Low	Moderate	Low	Moderate	Moderate	Moderate
Singh et al. (2013)	Moderate	Moderate	Low	Low	Low	Moderate	Moderate	Moderate
Yilmaz et al. (2013)	Moderate	Moderate	Low	Moderate	Low	Moderate	Moderate	Moderate
Jirge et al. (2014)	Moderate	Moderate	Low	Moderate	Low	Moderate	Moderate	Moderate
Xu et al. (2014)	Low	Moderate	Low	Low	Low	Moderate	Moderate	Moderate
Zangmo et al. (2014)	Moderate	Moderate	Low	Low	Low	Moderate	Moderate	Moderate
Tsui et al. (2015)	Moderate	Moderate	Low	Low	Low	Moderate	Moderate	Moderate
Vlahos et al. (2015)	Low	Moderate	Low	Low	Low	Moderate	Moderate	Moderate
Hu et al. (2017)	Low	Moderate	Low	Low	Low	Moderate	Moderate	Moderate
Chern et al. (2018)	Moderate	Moderate	Low	Low	Low	Moderate	Moderate	Moderate
Al-Turki et al. (2018)	Low	Moderate	Low	Low	Low	Moderate	Moderate	Moderate
Chen et al. (2019)	Moderate	Moderate	Low	Low	Low	Moderate	Moderate	Moderate
Ozcil et al. (2020)	Moderate	Moderate	Low	Moderate	Low	Moderate	Moderate	Moderate

Table S5. Meta-analyses of patients treated with DHEA or not.

Outcomes	No	WMD/RR	95%CI	*P* value for outcomes	heterogeneity		Range
					I^2, %	*P* value	
**AFC**							
Overall studies	10	0.96	0.48 to 1.44	<0.001	74.3	<0.001	1.68-8.76
RCTs	4	1.18	0.17 to 2.19	0.022	87.6	<0.001	
Non-RCTs	6	0.79	0.32 to 1.27	0.001	45.6	0.102	
**FSH, IU/L**							
Overall studies	13	-2.03	-3.10 to -0.95	<0.001	89.4	<0.001	7.6-14.7
RCTs	4	-1.99	-2.52 to -1.46	<0.001	0	0.571	
Non-RCTs	9	-2.22	-3.8 to -0.64	0.006	92.8	<0.001	
**AMH, ng/mL**							
Overall studies	13	0.34	0.17 to 0.51	<0.001	95	<0.001	0.65-3.8
RCTs	5	0.1	-0.14 to 0.34	0.416	71.6	0.007	
Non-RCTs	8	0.48	0.25 to 0.71	<0.001	96.5	<0.001	
**Total doses of Gn, IU**							
Overall studies	20	-211.87	-336.9 to -86.85	0.001	92.3	<0.001	2104-3920
RCTs	9	-382.29	-644.82 to -119.76	0.004	93.6	<0.001	
Non-RCTs	11	-45.72	-200.72 to 109.28	0.563	83.3	<0.001	
**Days of stimulation, days**							
Overall studies	19	-0.36	-0.62 to -0.10	0.007	80.4	<0.001	8.75-13.4
RCTs	8	-0.90	-1.34 to -0.47	<0.001	73.2	<0.001	
Non-RCTs	11	0.04	-0.14 to 0.23	0.663	39.7	0.084	
**E_2 level on the day of hCG, pg/mL**							
Overall studies	25	88.43	45.15 to 131.71	<0.001	96.2	<0.001	575-5082
RCTs	13	-33.21	-222.59 to 156.17	0.731	97.4	<0.001	

Non-RCTs	12	65.84	25.70 to 105.99	0.001	93.4	<0.001	
**Endometrial thickness, mm**							
Overall studies	13	0.58	-0.01 to 1.18	0.056	83.4	<0.001	8.76-12.05
RCTs	6	0.73	-0.05 to 1.52	0.067	82.0	<0.001	
Non-RCTs	7	0.41	-0.33 to 1.16	0.274	71.0	0.002	
**Number of oocytes retrieved**							
Overall studies	25	0.99	0.41 to 1.56	0.001	98.5	<0.001	1.9-8.3
RCTs	10	0.76	-0.20 to 1.72	0.123	93.9	<0.001	
Non-RCTs	15	1.14	0.16 to 2.12	0.023	99.0	<0.001	
**Number of oocytes transferred**							
Overall studies	20	0.27	0.01 to 0.52	0.040	97.3	<0.001	0.8-2.8
RCTs	7	0.19	-0.15 to 0.53	0.274	87.2	<0.001	
Non-RCTs	13	0.31	-0.01 to 0.64	0.057	98.1	<0.001	
**Clinical pregnancy rate**							
Overall studies	27	1.34	1.17 to 1.55	<0.001	0	0.511	/
RCTs	13	1.18	0.98 to 1.41	0.081	0	0.83	
Non-RCTs	14	1.63	1.30 to 2.05	<0.001	0.8	0.439	
**Live birth rate**							
Overall studies	12	1.86	1.21 to 2.86	0.005	57.7	0.007	/
RCTs	6	1.59	0.87 to 2.93	0.134	64.5	0.015	
Non-RCTs	6	2.26	1.25 to 4.10	0.007	42.4	0.123	
**Miscarriage rate**							
Overall studies	13	0.51	0.36 to 0.72	<0.001	4.9	0.397	/
RCTs	6	0.46	0.29 to 0.73	0.001	0	0.752	
Non-RCTs	7	0.59	0.35 to 1.00	0.048	44.4	0.109	

Table S6. Univariable meta-regression analyses for outcomes with significant difference.

	FSH			AMH			Days of stimulation			E_2 level on the day of hCG administration		
Variable	b	95%CI	*P*	b	95%CI	*P*	b	95%CI	*P*	b	95%CI	*P*
**Individual-level**												
Baseline age, y	-0.04	-0.92 to 0.84	0.922	-0.12	-0.33 to 0.09	0.230	0.009	-0.16 to 0.18	0.903	-25.44	-146.79 to 95.91	0.669
Baseline BMI, kg/m^2	0.46	-0.82 to 1.73	0.425	-0.03	-0.24 to 0.18	0.743	0.01	-0.21 to 0.24	0.902	-22.24	-166.02 to 121.54	0.746
Basal FSH, IU/L	-0.944	-1.62 to -0.25	**0.014**	0.14	-0.10 to 0.37	0.200	-0.03	-0.22 to 0.17	0.776	-4.28	-140.05 to 131.50	0.948
Basal E_2, pg/mL	0.001	-0.05 to 0.05	0.942	0.0004	-0.006 to 0.006	0.838	0.04	-0.002 to 0.09	0.057	-0.63	-1.48 to 0.21	0.122
AMH, ng/mL	2.99	-1.12 to 7.10	0.125	-0.60	-1.15 to -0.06	**0.035**	-0.30	-1.49 to 0.89	0.585	222.35	30.15 to 414.55	**0.027**
AFC	-0.03	-1.06 to 0.99	0.939	0.016	-0.36 to 0.39	0.919	0.02	-0.31 to 0.35	0.913	104.08	2.30 to 205.85	**0.046**
Duration of infertility, y	-0.35	-1.97 to 1.27	0.542	0.22	-0.42 to 0.86	0.396	-0.13	-0.47 to 0.21	0.422	39.18	-112.54 to 190.91	0.586
**Study-level**												
Publication year	-0.15	-0.64 to 0.35	0.518	-0.08	-0.29 to 0.12	0.396	-0.003	-0.13 to 0.12	0.959	20.97	-61.98 to 103.93	0.606
Study design												
RCTs	1[Ref.]	NA	NA	1[Ref.]	NA	NA	1[Ref.]	NA	NA	1[Ref.]	NA	NA
Case-control	1.09	-4.72 to 6.90	0.685	0.12	-1.08 to 1.32	0.832	0.95	0.31 to 1.60	**0.007**	600.71	-216.16 to 1417.58	0.141
Self-control	-0.96	-5.40 to 3.49	0.642	0.65	-0.25 to 1.55	0.140	0.82	0.09 to 1.55	**0.029**	268.04	-304.50 to 840.58	0.342
Sample size	0.007	-0.043 to 0.06	0.761	-0.002	-0.01 to 0.009	0.660	0.001	-0.0007 to 0.003	0.227	-0.22	-1.80 to 1.36	0.778
Area, continent												
East Asia	1[Ref.]	NA	NA	1[Ref.]	NA	NA	1[Ref.]	NA	NA	1[Ref.]	NA	NA
South Asia	-1.41	-7.56 to 4.74	0.612	1.24	0.60 to 1.88	**0.002**	0.06	-1.02 to 1.14	0.904	894.69	-158.68 to 1948.06	0.091
West Asia	0.61	-5.11 to 6.33	0.812	-0.05	-0.57 to 0.47	0.832	0.64	-0.61 to 1.90	0.291	260.01	-414.38 to 934.40	0.430
Europe	2.41	-3.90 to 8.72	0.405	-0.30	-0.85 to 0.24	0.238	-0.19	-1.56 to 1.19	0.776	434.37	-429.44 to 1298.19	0.306
Africa	0.44	-7.39 to 8.28	0.899	-0.06	-0.72 to 0.59	0.831	-0.69	-1.90 to 0.53	0.245	525.65	-488.23 to 1539.53	0.291
America	NA	NA	NA	NA	NA	NA	NA	NA	NA	770.43	-620.28 to 2161.14	0.261
Duration of DHEA												
<3 months	1[Ref.]	NA	NA	1[Ref.]	NA	NA	1[Ref.]	NA	NA	1[Ref.]	NA	NA
=3 months	0.25	-4.78 to 5.29	0.913	-0.11	-0.75 to 0.53	0.705	-0.13	-1.07 to 0.80	0.768	-445.20	-995.87 to 10648	0.108
>3 months	-1.42	-5.96 to 2.25	0.338	0.96	0.15 to 1.77	0.025	1.20	1.09 to 1.50	0.744	295.78	-544.41 to 1135.97	0.473

Figure S1. Results of quality assessment using ROB2 for RCTs.

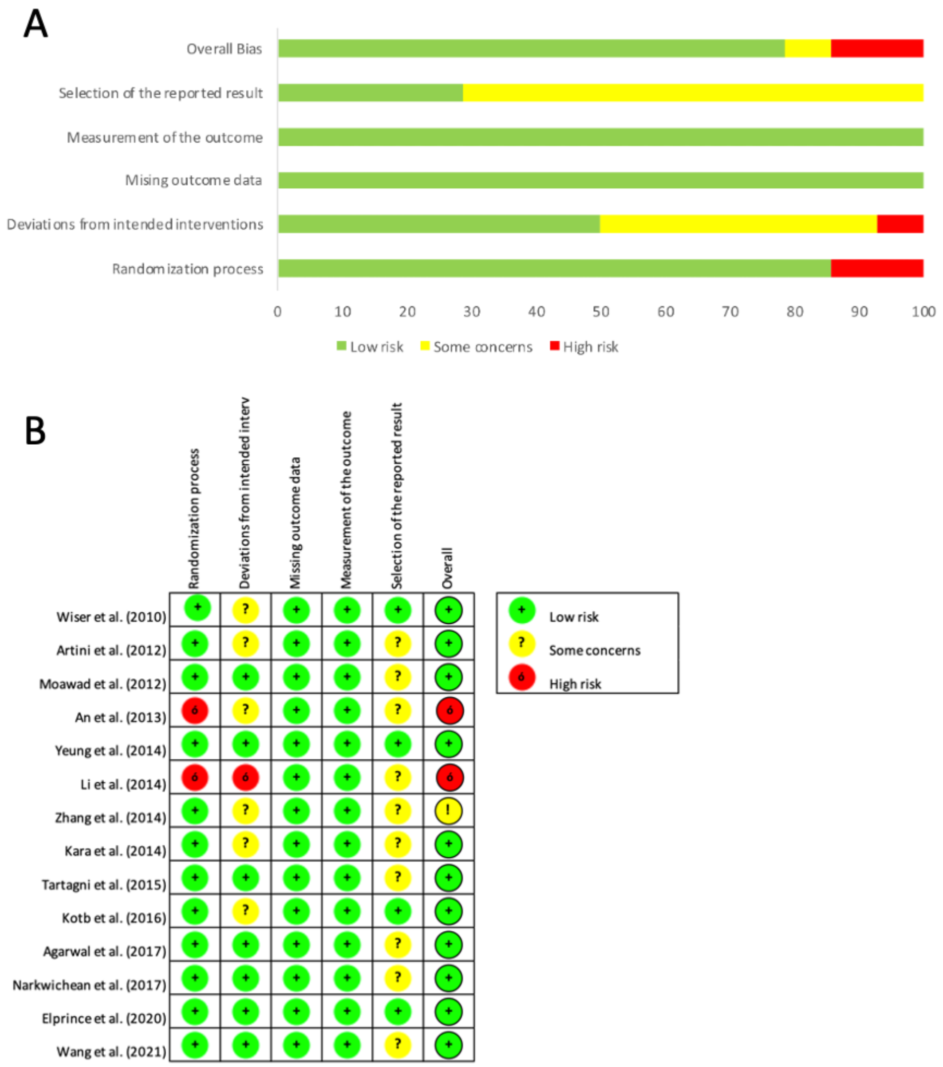

Figure S2. Funnel plots of the studies to assess publishing bias for **(A)** AFC (P_{Egger}=0.132); **(B)** AMH (P_{Egger}=0.009); **(C)** FSH (P_{Egger}=0.973); **(D)** Total doses of gonadotropin (P_{Egger}=0.021); **(E)** Days of stimulation (P_{Egger}=0.084); **(F)** E_2 level on the day of hCG (P_{Egger}=0.218); **(G)** Endometrial thickness (P_{Egger}=0.529); **(H)** Number of oocytes retrieved (P_{Egger}=0.002); **(I)** Number of oocytes transferred (P_{Egger}=0.044); **(J)** Clinical pregnancy rate of IVF (P_{Egger}=0.106); **(K)** Live birth rate (P_{Egger}=0.453); **(L)** Miscarriage rate(P_{Egger}=0.435).

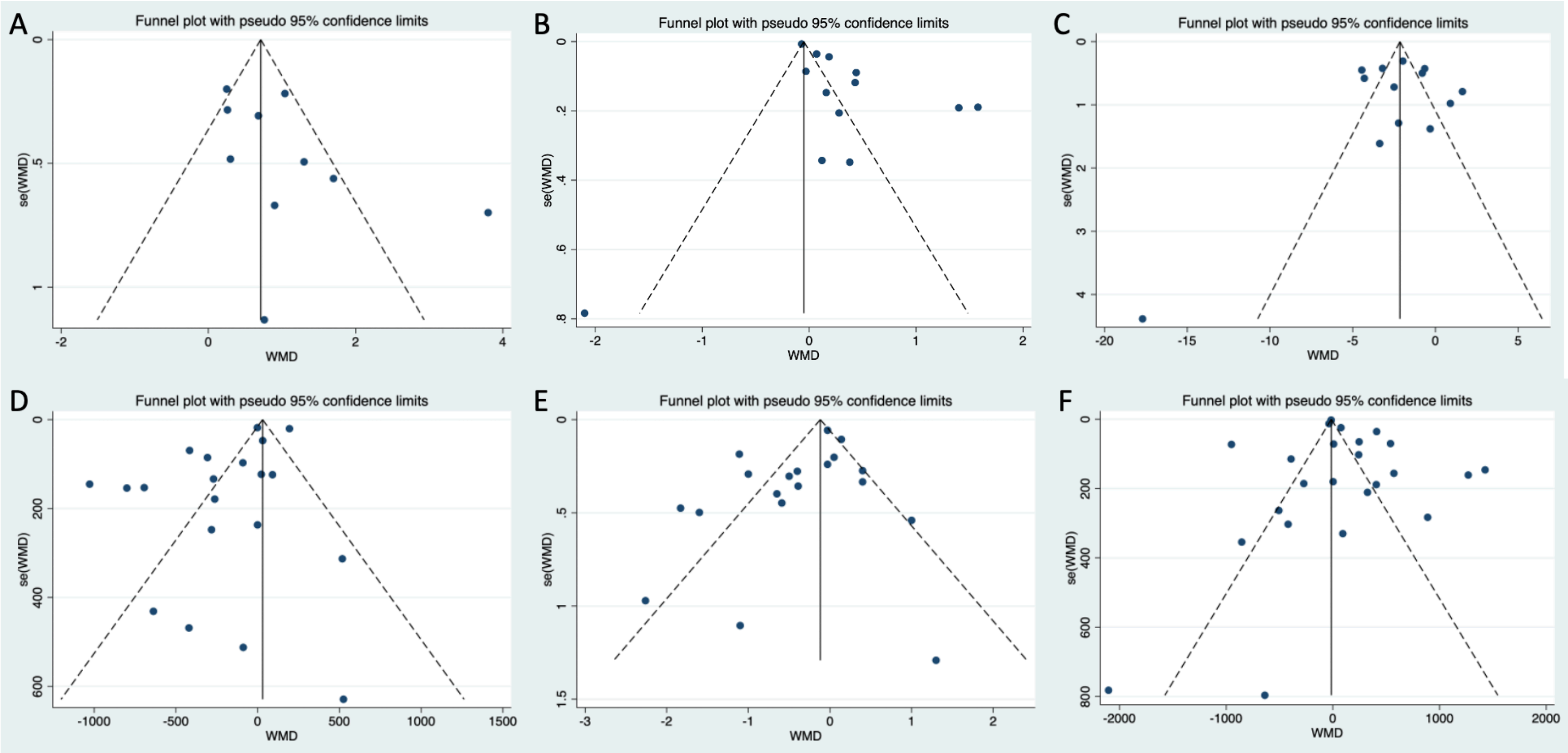

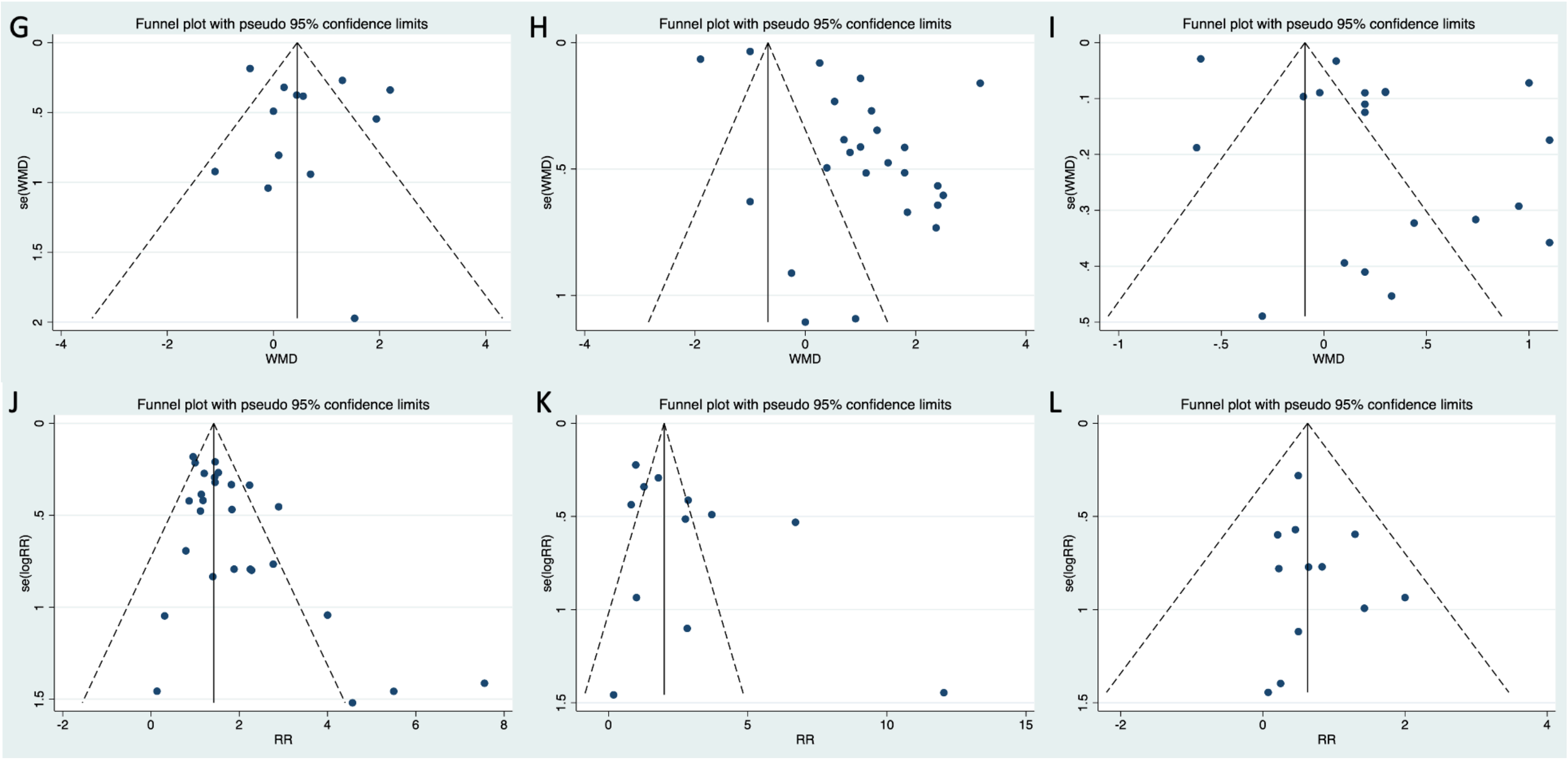
G
Funnel plot with pseudo 95% confidence limits
se(WMD)
WMD
H
Funnel plot with pseudo 95% confidence limits
se(WMD)
WMD
I
Funnel plot with pseudo 95% confidence limits
se(WMD)
WMD
J
Funnel plot with pseudo 95% confidence limits
se(logRR)
RR
K
Funnel plot with pseudo 95% confidence limits
se(logRR)
RR
L
Funnel plot with pseudo 95% confidence limits
se(logRR)
RR

Figure S3. The subgroup meta-analysis by type of study design for the outcome of days of stimulation in poor responders undergoing IVF treatment.

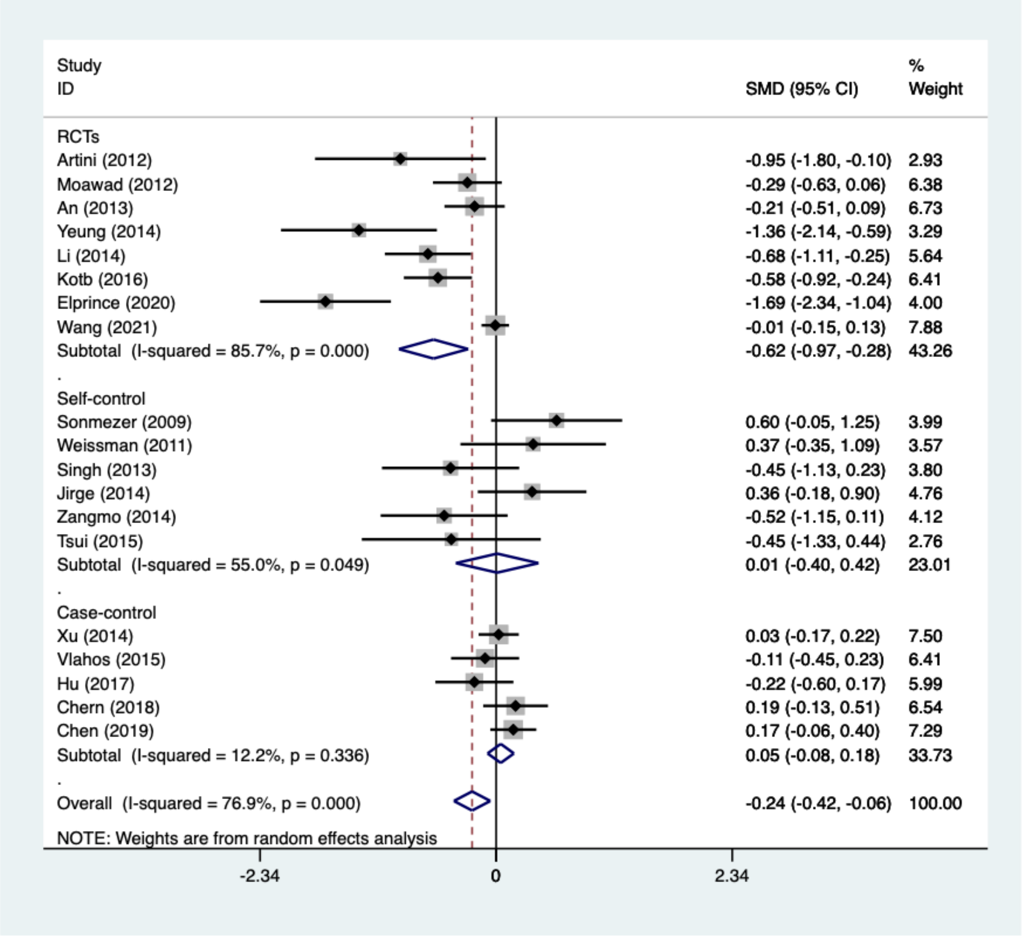

References:

1. Wiser A, Gonen O, Ghetler Y, Shavit T, Berkovitz A, Shulman A. Addition of dehydroepiandrosterone (DHEA) for poor-responder patients before and during IVF treatment improves the pregnancy rate: a randomized prospective study. *Hum Reprod* (2010) 25:2496-500. doi: 10.1093/humrep/deq220
2. Artini PG, Simi G, Ruggiero M, Pinelli S, Di Berardino OM, Papini F, et al. DHEA supplementation improves follicular microenviroment in poor responder patients. *Gynecol Endocrinol* (2012) 28:669-73. doi: 10.3109/09513590.2012.705386
3. Moawad A, Shaeer M. Long-term androgen priming by use of dehydroepiandrosterone (DHEA) improves IVF outcome in poor-responder patients. A randomized controlled study. *Middle East Fertil Soc J* (2012) 17:137-42. doi: 10.1007/s13224-016-0941-8
4. An J, Wang Y, Ni YL, Chai SM. Application of dehydroepiandrosterone (DHEA) supplementation in in vitro fertilization and embryo transfer cycles. *Reprod Contracep* (2013) 33:89-92. doi: 10.1007/s13224-016-0941-8
5. Yeung TW, Chai J, Li RH, Lee VC, Ho PC, Ng EH. A randomized, controlled, pilot trial on the effect of dehydroepiandrosterone on ovarian response markers, ovarian response, and in vitro fertilization outcomes in poor responders. *Fertil Steril* (2014) 102:108-15 e1. doi: 10.1016/j.fertnstert.2014.03.044
6. Li J, Ren CE, Zhang AQ, Du T. Effect of dehydroepiandrosterone (DHEA) supplementation on IVF-ET of patients with diminished ovarian reserve (DOR). *J Liaoning Medical University* (2014) 35:51-3. doi: 10.1136/bmj.i4919
7. Zhang HH, Xu PY, Wu J, Zou WW, Xu XM, Cao XY, et al. Dehydroepiandrosterone improves follicular fluid bone morphogenetic protein-15 and accumulated embryo score of infertility patients with diminished ovarian reserve undergoing in vitro fertilization: a randomized controlled trial. *J Ovarian Res* (2014) 7:93. doi: 10.1186/s13048-014-0093-3
8. Kara M, Aydin T, Aran T, Turktekin N, Ozdemir B. Does dehydroepiandrosterone supplementation really affect IVF-ICSI outcome in women with poor ovarian reserve? *Eur J Obstet Gynecol Reprod Biol* (2014) 173:63-5. doi: 10.1016/j.ejogrb.2013.11.008
9. Tartagni M, Cicinelli MV, Baldini D, Tartagni MV, Alrasheed H, DeSalvia MA, et al. Dehydroepiandrosterone decreases the age-related decline of the in vitro fertilization outcome in women younger than 40 years old. *Reprod Biol Endocrinol* (2015) 13:18. doi: 10.1186/s12958-015-0014-3
10. Kotb MM, Hassan AM, AwadAllah AM. Does dehydroepiandrosterone improve pregnancy rate in women undergoing IVF/ICSI with expected poor ovarian response according to the Bologna criteria? A randomized controlled trial. *Eur J Obstet Gynecol Reprod Biol* (2016) 200:11-5. doi: 10.1016/j.ejogrb.2016.02.009
11. Agarwal R, Shruthi R, Radhakrishnan G, Singh A. Evaluation of Dehydroepiandrosterone Supplementation on Diminished Ovarian Reserve: A Randomized, Double-Blinded, Placebo-Controlled Study. *J Obstet Gynaecol India* (2017) 67:137-42. doi: 10.1007/s13224-016-0941-8

12. Narkwichean A, Maalouf W, Baumgarten M, Polanski L, Raine-Fenning N, Campbell B, et al. Efficacy of Dehydroepiandrosterone (DHEA) to overcome the effect of ovarian ageing (DITTO): A proof of principle double blinded randomized placebo controlled trial. *Eur J Obstet Gynecol Reprod Biol* (2017) 218:39-48. doi: 10.1016/j.ejogrb.2017.09.006
13. Elprince M, Kishk EA, Metawie OM, Albiely MM. Ovarian stimulation after dehydroepiandrosterone supplementation in poor ovarian reserve: a randomized clinical trial. *Arch Gynecol Obstet* (2020) 302:529-34. doi: 10.1007/s00404-020-05603-5
14. Wang Z, Yang A, Bao H, Wang A, Deng X, Xue D, et al. Effect of dehydroepiandrosterone administration before in vitro fertilization on the live birth rate in poor ovarian responders according to the Bologna criteria: A randomised controlled trial. *BJOG* (2022) 129:1030-8. doi: 10.1111/1471-0528.17045
15. Barad D, Gleicher N. Effect of dehydroepiandrosterone on oocyte and embryo yields, embryo grade and cell number in IVF. *Hum Reprod* (2006) 21:2845-9. doi: 10.1093/humrep/del254
16. Barad D, Brill H, Gleicher N. Update on the use of dehydroepiandrosterone supplementation among women with diminished ovarian function. *J Assist Reprod Genet* (2007) 24:629-34. doi: 10.1007/s10815-007-9178-x
17. Sönmezer M, Ozmen B, Cil AP, Ozkavukçu S, Taşçi T, Olmuş H, et al. Dehydroepiandrosterone supplementation improves ovarian response and cycle outcome in poor responders. *Reprod Biomed Online* (2009) 19:508-13. doi: 10.1016/j.rbmo.2009.06.006
18. Weissman A, Horowitz E, Ravhon A, Golan A, Levran D. Dehydroepiandrosterone supplementation increases baseline follicular phase progesterone levels. *Gynecol Endocrinol* (2011) 27:1014-7. doi: 10.3109/09513590.2011.569611
19. Fusi FM, Ferrario M, Bosisio C, Arnoldi M, Zanga L. DHEA supplementation positively affects spontaneous pregnancies in women with diminished ovarian function. *Gynecol Endocrinol* (2013) 29:940-3. doi: 10.3109/09513590.2013.819087
20. Hyman JH, Margalioth EJ, Rabinowitz R, Tsafrir A, Gal M, Alerhand S, et al. DHEA supplementation may improve IVF outcome in poor responders: a proposed mechanism. *Eur J Obstet Gynecol Reprod Biol* (2013) 168:49-53. doi: 10.1016/j.ejogrb.2012.12.017
21. Singh N, Zangmo R, Kumar S, Roy KK, Sharma JB, Malhotra N, et al. A prospective study on role of dehydroepiandrosterone (DHEA) on improving the ovarian reserve markers in infertile patients with poor ovarian reserve. *Gynecol Endocrinol* (2013) 29:989-92. doi: 10.3109/09513590.2013.824957
22. Yilmaz N, Uygur D, Inal H, Gorkem U, Cicek N, Mollamahmutoglu L. Dehydroepiandrosterone supplementation improves predictive markers for diminished ovarian reserve: serum AMH, inhibin B and antral follicle count. *Eur J Obstet Gynecol Reprod Biol* (2013) 169:257-60. doi: 10.1016/j.ejogrb.2013.04.003
23. Jirge PR, Chougule SM, Gavali VG, Bhomkar DA. Impact of dehydroepiandrosterone on clinical outcome in poor responders: A pilot study in women undergoing in vitro fertilization, using bologna criteria. *J Hum Reprod Sci* (2014) 7:175-80. doi: 10.4103/0974-1208.142477
24. Xu B, Li Z, Yue J, Jin L, Li Y, Ai J, et al. Effect of dehydroepiandrosterone administration in patients with poor ovarian response according to the Bologna criteria. *PLoS One* (2014) 9:e99858. doi: 10.1371/journal.pone.0099858

25. Zangmo R, Singh N, Kumar S, Vanamail P, Tiwari A. Role of dehydroepiandrosterone in improving oocyte and embryo quality in IVF cycles. *Reprod Biomed Online* (2014) 28:743-7. doi: 10.1016/j.rbmo.2014.01.019
26. Tsui KH, Lin LT, Chang R, Huang BS, Cheng JT, Wang PH. Effects of dehydroepiandrosterone supplementation on women with poor ovarian response: A preliminary report and review. *Taiwan J Obstet Gynecol* (2015) 54:131-6. doi: 10.1016/j.tjog.2014.07.007
27. Vlahos N, Papalouka M, Triantafyllidou O, Vlachos A, Vakas P, Grimbizis G, et al. Dehydroepiandrosterone administration before IVF in poor responders: a prospective cohort study. *Reprod Biomed Online* (2015) 30:191-6. doi: 10.1016/j.rbmo.2014.10.005
28. Hu Q, Hong L, Nie M, Wang Q, Fang Y, Dai Y, et al. The effect of dehydroepiandrosterone supplementation on ovarian response is associated with androgen receptor in diminished ovarian reserve women. *J Ovarian Res* (2017) 10:32. doi: 10.1186/s13048-017-0326-3
29. Chern CU, Tsui KH, Vitale SG, Chen SN, Wang PH, Cianci A, et al. Dehydroepiandrosterone (DHEA) supplementation improves in vitro fertilization outcomes of poor ovarian responders, especially in women with low serum concentration of DHEA-S: a retrospective cohort study. *Reprod Biol Endocrinol* (2018) 16:90. doi: 10.1186/s12958-018-0409-z
30. Al-Turki HA. Dehydroepiandrosterone supplementation in women undergoing assisted reproductive technology with poor ovarian response. A prospective case-control study. *J Int Med Res* (2018) 46:143-9. doi: 10.1177/0300060517720005
31. Chen SN, Tsui KH, Wang PH, Chern CU, Wen ZH, Lin LT. Dehydroepiandrosterone Supplementation Improves the Outcomes of in vitro Fertilization Cycles in Older Patients With Diminished Ovarian Reserve. *Front Endocrinol (Lausanne)* (2019) 10:800. doi: 10.3389/fendo.2019.00800
32. Ozcil MD. Dehydroepiandrosterone supplementation improves ovarian reserve and pregnancy rates in poor responders. *Eur Rev Med Pharmacol Sci* (2020) 24:9104-11. doi: 10.26355/eurrev_202009_22856
